# Transcriptomic analysis of regulatory pathways involved in female reproductive physiology of *Rhodnius prolixus* under different nutritional states

**DOI:** 10.1038/s41598-020-67932-4

**Published:** 2020-07-10

**Authors:** Jimena Leyria, Ian Orchard, Angela B. Lange

**Affiliations:** Department of Biology, University of Toronto Mississauga, Mississauga, ON Canada

**Keywords:** Reproductive biology, Transcriptomics, Molecular biology, Physiology

## Abstract

The triatomine *Rhodnius prolixus*, a vector of the etiological agent of Chagas disease, has long been used as model to understand important aspects of insect physiology. Despite this history, the impact of the nutritional state on regulatory pathways associated with reproductive performance in triatomines has never been studied. The insulin-like peptide/target of rapamycin (ILP/ToR) signaling pathway is typically responsible for detecting and interpreting nutrient levels. Here, we analyzed transcriptomes from the central nervous system, fat bodies and ovaries of adult females in unfed and fed conditions, with a focus on the ILP/ToR signaling. The results show an up-regulation of transcripts involved in ILP/ToR signaling in unfed insects. However, we demonstrate that this signaling is only activated in tissues from fed insects. Moreover, we report that FoxO (forkhead box O) factor, which regulates longevity via ILP signaling, is responsible for the up-regulation of transcripts related with ILP/ToR signaling in unfed insects. As a consequence, we reveal that unfed females are in a sensitized state to respond to an increase of ILP levels by rapidly activating ILP/ToR signaling. This is the first analysis that correlates gene expression and protein activation of molecules involved with ILP/ToR signaling in *R. prolixus* females in different nutritional states.

## Introduction

The triatomine *Rhodnius prolixus* is a blood-feeding insect which, because of anthropophily, rapid development cycle, intense passive dispersion and high susceptibility to infection by *Trypanosoma cruzi,* is one of the most important vectors of Chagas disease^1^. Currently, this disease affects approximately 8 million people and migration from endemic to non-endemic regions has made the disease more global with approximately 350,000 infected carriers^2^. The lack of specific treatments in the chronic phase, and the absence of vaccines for prevention, make insect control the main strategy to reduce Chagas’ prevalence^3^. Moreover, over the past century, *R. prolixus* has been the subject of intense investigation, which has contributed to our understanding of important aspects of metabolism, endocrinology and physiology of all insect models^4^. Despite these studies, many aspects of the biology of this species remain to be elucidated.

In insects, reproduction involves egg production and consequently, if the female is mated, a new generation. In this sense, oviparous females must drive, with extraordinary effectiveness, the conversion of nutritional resources into eggs. Yolk deposition, referred to as vitellogenesis, is characterized by a massive synthesis of yolk protein precursors (YPPs), lipids and carbohydrates by the fat body, an organ analogous to liver and adipose tissue in mammals^5^. These nutrients are released into the hemolymph and then internalized by oocytes to promote egg growth. Vitellogenesis is controlled by hormonal signaling that involves neuropeptides, juvenile hormones (JH) and ecdysteroids^5^. Specific neuropeptides which promote egg production are the insulin-like peptides (ILPs)^6^. Insect ILPs are analogous to both insulin and insulin growth factor (IGF) of vertebrates, and so far, it is understood that these act by a conserved insulin signaling pathway^7^. It has been shown in some insects that when the female reaches an adequate nutritional state, the ILPs are secreted into the hemolymph^7^. Binding of ILPs to the insulin receptor (InR) activates the insulin receptor substrate proteins (IRS), promoting phosphatidylinositol 3-kinase (PI3K) expression and the production of phosphatidylinositol trisphosphate (PIP3). A key downstream effector of PIP3 is a serine/threonine-protein kinase, Akt, which in turn phosphorylates a series of mediators such as forkhead box O transcription factor (FoxO) and glycogen synthase kinase (GSK)^8^. The insulin pathway is responsive to nutrient intake through the target of rapamycin (ToR) signaling. ToR is a serine/threonine kinase that is highly conserved in most eukaryotes^9^. Targets for mToR are proteins involved in controlling mRNA translation, including the ribosomal protein S6 kinases (p70S6K) and the initiation factor 4E-binding proteins (4E-BPs)^9^. Together, ILP/ToR signaling represents a nutritional sensing mechanism and plays a crucial role in determining the tradeoff between reproductive success and survival in some insect species^6^. Recently, in juvenile stages of *R. prolixus,* we identified ILP, IGF and InR^10–12^. ILP is only produced by a small group of medial neurosecretory cells in the brain. In contrast, IGF and InR are expressed in a variety of tissues, with the highest transcript levels found in the fat body and central nervous system (CNS), respectively. Overall, these proteins act as modulators of lipid and carbohydrate metabolism, probably via sensing the requirement and/or presence of nutrients in the hemolymph according to the physiological state of the insect^10–12^. The relationship between the ILP/ToR signaling and reproductive performance in triatomines has never been studied. In this context, *R. prolixus* represents a perfect model to study events related to insect reproduction since it is possible to define the unfed state and activate the reproductive process by providing a blood meal.

In the last decade, next-generation sequencing (RNA-seq) has enabled transcript profile analyses. Here, we perform a transcriptome analysis focusing on different regulatory pathways associated with nutritional state. This is the first analysis to correlate gene expression and protein activation involved with ILP/ToR signaling in *R. prolixus* females in different nutritional conditions.

## Results and discussion

### Illumina sequencing and read assembly

RNA-seq metrics from *R. prolixus* transcriptomes for CNS, ovaries (OV) and fat bodies (FB) under both unfed condition (UFC) and fed condition (FC) are summarized in Table 1. The data quality control showed indices expected to advance towards a high quality transcriptome analysis. The quantity of total mapped reads with the reference genome, including those multiple and uniquely mapped, and percentages of clean reads are also shown (Table 1).

**Table 1:** Summary of RNA-seq metrics from *R. prolixus* transcriptomes for CNS, ovaries and fat bodies under both fed and unfed conditions.

Sample name	Raw reads	Clean reads	Raw bases	Clean bases	Error rate (%)	Q20 (%)	Q30 (%)	GC content (%)	Total mapped	Multiple mapped	Uniquely mapped
FB_FC1	49,056,988	47,978,984	7.4G	7.2G	0.03	97.45	92.97	38.83	45,263,367 (94.34%)	8,772,445 (18.28%)	36,490,922 (76.06%)
FB_FC2	57,872,080	56,052,124	8.7G	8.4G	0.03	97.39	92.88	38.16	52,821,697 (94.24%)	12,155,625 (21.69%)	40,666,072 (72.55%)
FB_FC3	45,020,482	43,727,054	6.8G	6.6G	0.03	97.31	92.71	38.72	40,072,041 (91.64%)	6,265,951 (14.33%)	33,806,090 (77.31%)
FB_UFC1	38,034,808	36,589,736	5.7G	5.5G	0.03	97.64	93.3	37.55	33,952,082 (92.79%)	6,587,693 (18%)	27,364,389 (74.79%)
FB_UFC2	43,020,456	42,041,810	6.5G	6.3G	0.03	97.73	93.41	37.35	40,040,549 (95.24%)	9,447,711 (22.47%)	30,592,838 (72.77%)
FB_UFC3	44,053,400	43,233,418	6.6G	6.5G	0.03	97.87	93.57	37.58	40,592,581 (93.89%)	8,453,828 (19.55%)	32,138,753 (74.34%)
CNS_FC1	40,657,664	39,529,300	6.1G	5.9G	0.03	97.42	92.88	35.95	36,566,622 (92.51%)	7,905,860 (20%)	28,660,762 (72.51%)
CNS_FC2	50,307,212	49,021,040	7.5G	7.4G	0.03	97.07	92.09	36.28	44,248,348 (90.26%)	7,073,404 (14.43%)	37,174,944 (75.83%)
CNS_FC3	38,743,954	37,935,224	5.8G	5.7G	0.03	97.84	93.51	35.99	34,975,847 (92.2%)	7,505,705 (19.79%)	27,470,142 (72.41%)
CNS_UFC1	44,272,692	42,992,970	6.6G	6.4G	0.03	97.21	92.4	36.67	39,481,851 (91.83%)	9,914,637 (23.06%)	29,567,214 (68.77%)
CNS_UFC2	38,282,234	37,345,712	5.7G	5.6G	0.03	97.29	92.53	35.92	34,177,059 (91.52%)	6,203,001 (16.61%)	27,974,058 (74.91%)
CNS_UFC3	45,396,058	44,395,532	6.8G	6.7G	0.03	97.9	93.7	36.45	41,172,365 (92.74%)	8,788,138 (19.8%)	32,384,227 (72.94%)
OV PV_FC1	36,463,170	35,554,174	5.5G	5.3G	0.03	97.53	93.05	36.68	33,300,407 (93.66%)	6,667,395 (18.75%)	26,633,012 (74.91%)
OV PV_FC2	40,990,242	39,839,314	6.1G	6G	0.03	97.21	92.39	36.92	36,530,770 (91.7%)	8,989,879 (22.57%)	27,540,891 (69.13%)
OV PV_FC3	41,493,598	40,357,828	6.2G	6.1G	0.03	97.99	93.86	36.41	37,803,688 (93.67%)	7,220,401 (17.89%)	30,583,287 (75.78%)
OV V_FC1	55,239,732	54,067,958	8.3G	8.1G	0.03	97.97	93.85	37.1	50,622,214 (93.63%)	8,837,180 (16.34%)	41,785,034 (77.28%)
OV V_FC2	53,087,318	52,044,590	8G	7.8G	0.03	98.06	94.07	37.16	48,331,585 (92.87%)	9,326,157 (17.92%)	39,005,428 (74.95%)
OV V_FC3	54,387,600	53,290,294	8.2G	8G	0.03	97.65	93.09	37.73	49,229,587 (92.38%)	9,015,656 (16.92%)	40,213,931 (75.46%)
OV_UFC1	48,976,014	46,993,620	7.3G	7G	0.03	97.8	93.45	36.17	44,187,120 (94.03%)	4,455,710 (9.48%)	39,731,410 (84.55%)
OV_UFC2	50,296,540	48,537,028	7.5G	7.3G	0.03	97.93	93.74	36.3	45,887,232 (94.54%)	4,834,351 (9.96%)	41,052,881 (84.58%)
OV_UFC3	57,460,666	55,196,208	8.6G	8.3G	0.03	97.83	93.46	36.37	52,061,906 (94.32%)	4,782,906 (8.67%)	47,279,000 (85.66%)

*Raw Reads*, the original sequencing reads counts; *Clean Reads*, number of reads after filtering; *Raw Bases*, raw reads number multiply read length, saved in G unit; *Clean Bases*, clean reads number multiply read length, saved in G unit; Error Rate: average sequencing error rate; *Q20*: percentages of bases whose correct base recognition rates are greater than 99% in total bases; *Q30*: percentages of bases whose correct base recognition rates are greater than 99.9% in total bases; *GC content*: percentages of G and C in total bases. *Total mapped*, total number of reads that can be mapped to the reference genome; *Multiple mapped*, number of reads that can be mapped to multiple sites in the reference genome; *Uniquely mapped*, number of reads that can be uniquely mapped to the reference genome.

### Total gene expression levels and co-expression analysis

**Figure 1:**
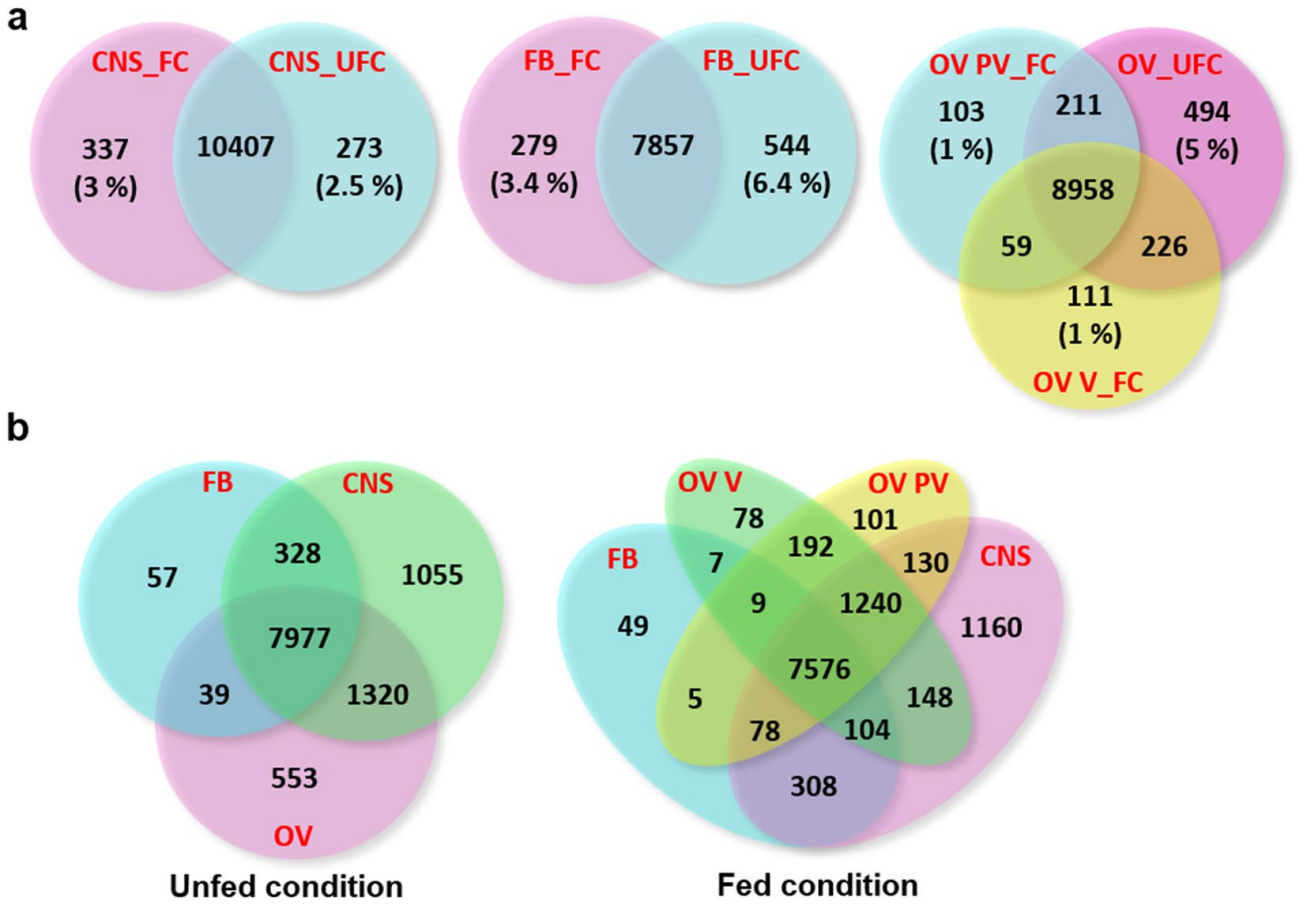
Gene co-expression. (**a**, **b**) Venn diagram presents the number of genes that are uniquely expressed within each sample (the percentage is shown), with the overlapping regions showing the number of genes that are expressed in two or more samples. (**a**) Tissue by different nutritional states; (**b**) same nutritional state comparing different tissues. CNS_FC, central nervous system post-feeding (FC, fed condition); CNS_UFC, central nervous system before of a blood meal (UFC, unfed condition); FB_FC, fat body in FC; FB_UFC, fat body in UFC; OV PV_FC, pre-vitellogenic ovariole (tropharium and immature oocytes) in FC; OV V_FC, vitellogenic ovariole (follicles containing mature oocytes) in FC; OV_UFC, ovariole in UFC.

Violin plots were used to compare total gene expression levels of each tissue under different nutritional states (Supplementary Fig. S1). The results show that the same tissues, even under different nutritional conditions, i.e. UFC and FC, have a gene population comparable in distribution and density. However, differences could be observed between CNS, FB and OV. Differentially expressed genes (DEG) were also screened and summarized in Venn diagrams (Fig. 1). The results reveal the existence of genes that are uniquely expressed within each tissue and each condition, along with genes with overlapping regions that are expressed in the same tissue in both nutritional states (Fig. 1a) or different tissues in the same nutritional state (Fig. 1b). It is important to keep in mind that the unique gene expression percentages in each tissue during a specific nutritional state are low relative to overall gene expression. These results suggest that the expression levels of the same genes (overlapping) could define the physiological/metabolic response of a tissue according to nutritional state and not the expression of unique genes.

To assess the relationship between gene expression profiles of the CNS, FB and OV in the different nutritional states, we performed unsupervised hierarchical clustering of our complete RNA-Seq transcriptome data (Supplementary Fig. S2). As expected, genes of the same tissue cluster together, even during different nutritional conditions, and genes of different tissues, i.e. CNS, FB and OV, cluster separately, consistent with the unique functions of each.

### Differentially expressed genes (DEG) analysis

**Figure 2:**
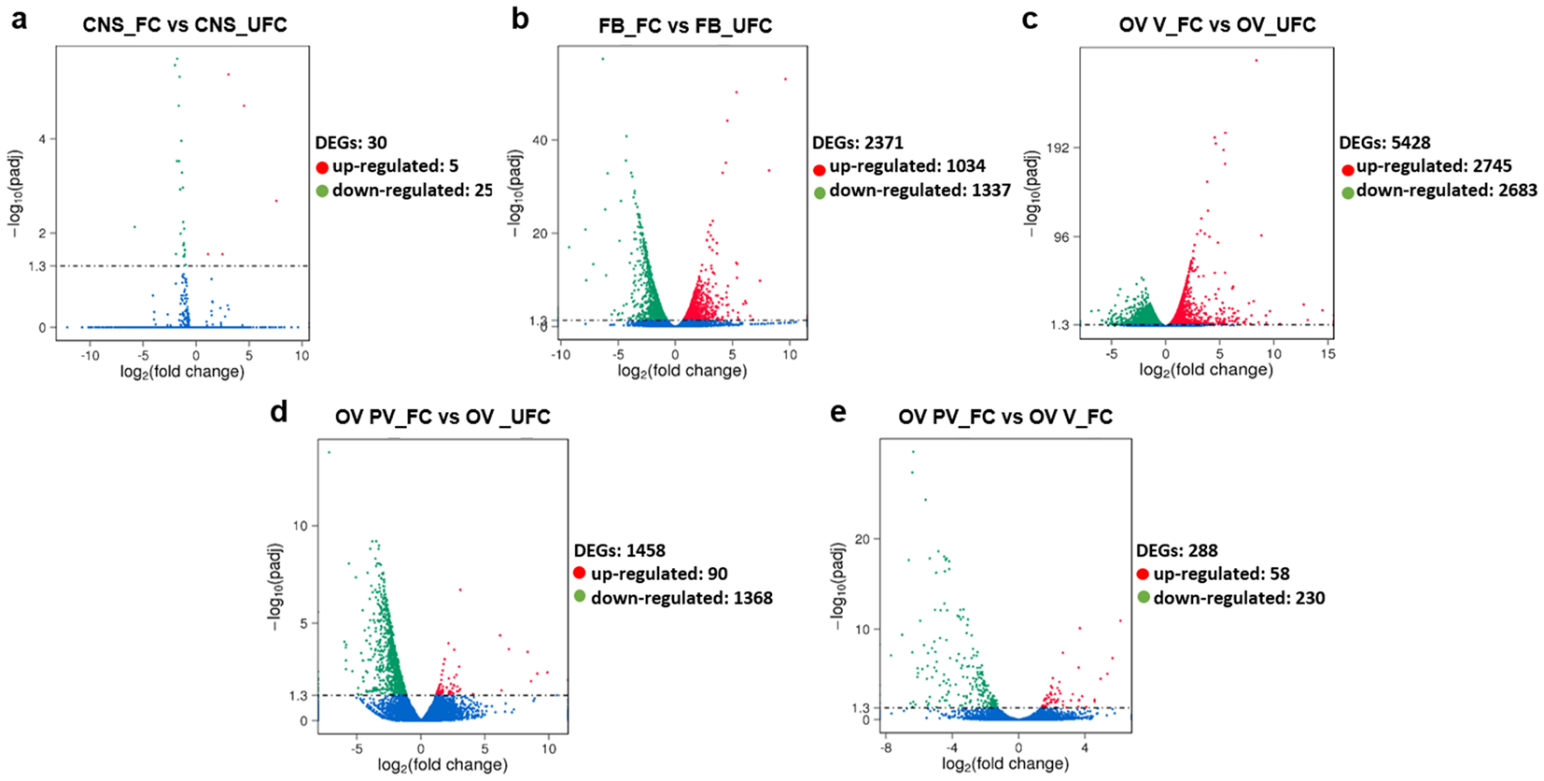
Screening of differentially expressed genes (DEG) by volcano plots. (**a**–**e**) The x-axis shows the fold change in gene expression of FC respect to UFC (log_2_ (fold change)) and the y-axis shows the statistical significance of the differences (− log_10_(padj)). Significantly up and down regulated genes are highlighted in red and green, respectively. Genes did not express differently are showed in blue. (**a**) CNS_FC versus CNS_UFC; (**b**) FB_FC versus FB_UFC; (**c**) OV V_FC versus OV_UFC; (**d**) OV PV_FC versus OV _UFC; (**e**), OV PV_FC versus OV V_FC.

Biological replicates were performed to demonstrate that the experiments are repeatable and to reveal differences in gene expression between the two nutritional states. A comparison among the 3 biological replicates of each nutritional state had a correlation coefficient (R^2^) close to 1 (most > 0.9) (Supplementary Fig. S3). With these data, we were confident to advance to the next analysis. First, DEG were screened and volcano plots were used to infer overall distribution (Fig. 2). Genes with *p*-adj < 0.05 were assigned as differentially expressed. The results show 30 DEG in CNS_FC versus CNS_UFC (0.27% of the total number of genes detected in CNS; Fig. 2a) and 2,371 genes in FB_FC versus FB_UFC (27.3% of the total number of genes detected in FB; Fig. 2b). To deepen the transcriptome analysis, the ovarioles from insects during the FC were further separated: (a) pre-vitellogenic ovariole (OV PV_FC), which include the tropharium and immature oocytes, and (b) vitellogenic ovariole (OV V_FC), which are the follicles containing mature oocytes. Ovarioles during UFC were used in their entirety (OV_UFC) (Supplementary Fig. S4). The results show 5,428 genes differentially expressed in OV V_FC versus OV_UFC (53.4% of the total number of genes detected in V; Fig. 2c) and 1,458 genes in OV PV_FC versus OV_UFC (14.35% of the total number of genes detected in OV; Fig. 2d). Overall, excluding the CNS, the results show significant changes in gene expression of each tissue when comparing the different nutritional states. It is important to highlight that different parts of an ovariole in the same nutritional condition, i.e. OV PV_FC and OV V_FC, have similar profiles of DEG (2.8% of the total number of genes detected in OV; Fig. 2e).

### ILP/ToR signaling analysis on CNS, fat bodies and ovaries

In insects, the availability of nutrients influences multiple signaling pathways. Nutrient depletion favors activation of processes involved in energy production, stress resistance and survival. On the other hand, in some species of insects, when the nutrients are abundant, ILP/ToR signaling is activated and cooperatively works with other pathways leading to successful reproduction^6^. For *R. prolixus*, specific genes related to ILP/ToR signaling were selected and analysed. As can be seen, the CNS is metabolically stable under both nutritional conditions, i.e. non-DEG were found (Table 2). Previously we reported for fifth instars of *R. prolixus* that no significant changes in ILP transcript expression was observed relative to feeding^10^. In *D. melanogaster,* ILP2 transcript levels were also insensitive to nutrient deprivation but the protein secretion and signaling activity depends on metabolic needs; thus ILP2 is present in a basal level in fed insects and accumulates upon starvation^13^. ILPs are mainly synthesized in the CNS, but in some insects several ILPs have been reported to be produced and released by the FB as well as by other tissues^6^. In adults of *R. prolixus*, ILP is exclusively expressed in the CNS with higher expression compared with other transcripts related to this signaling pathway (Table 2). Also, in fifth instars of *R. prolixus,* ILP transcript is expressed around 25,000 times higher in CNS than in other tissues^10^.

**Table 2:**
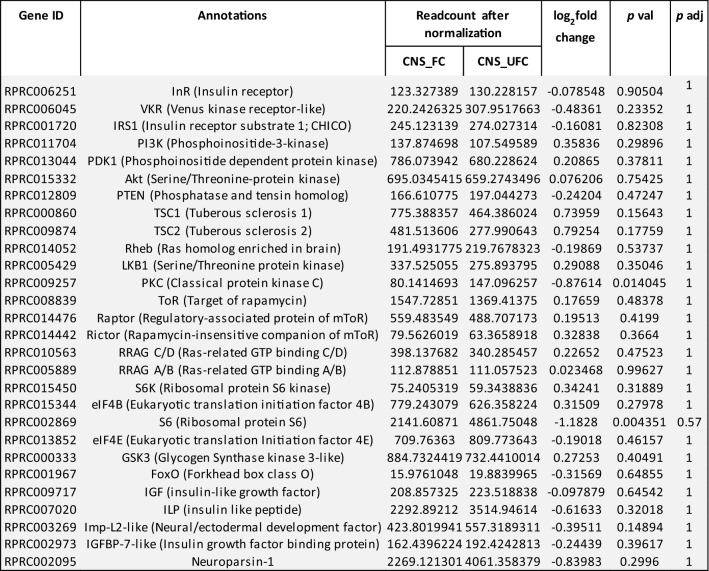
Expression profiles of genes involved in ILP/ToR signaling in CNS under fed and unfed conditions.

*Gray boxes*, genes which are not differentially expressed (non-DEG) between unfed and fed insects; Data shown as log_2_fold change of FC versus UFC. **Gene ID**: VectorBase code (the official gene number in the RproC3 genome assembly); **annotations**: the protein name we are assigning.

**Figure 3:**
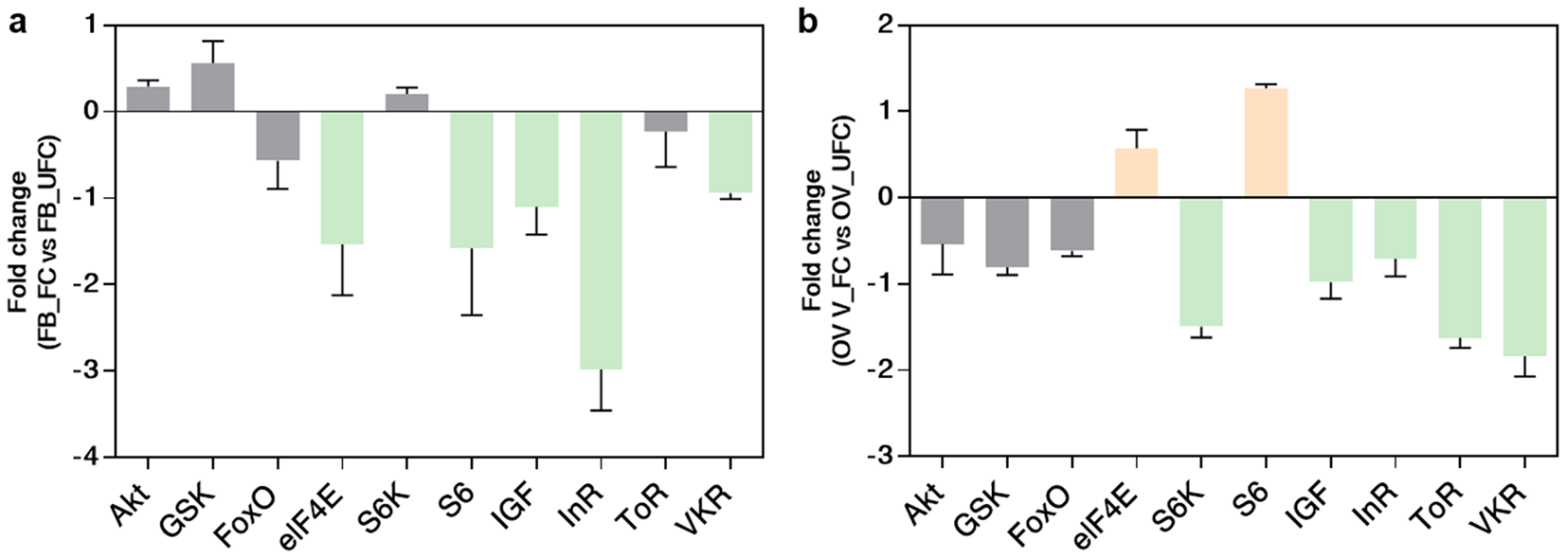
Verification of differentially expressed genes (DEG) by RT-qPCR to ILP/ToR signaling in fat body, FB (**a**) and ovaries, OV (**b**). The fold change of gene was calculated as transcript levels of fed insects/transcript levels of unfed insects (FC vs UFC). Values are expressed as mean ± SEM of 3 independent experiments. Graphs were performed using GraphPad Prism 7 (GraphPad Software, CA, USA, www.graphpad.com).

**Figure 4:**
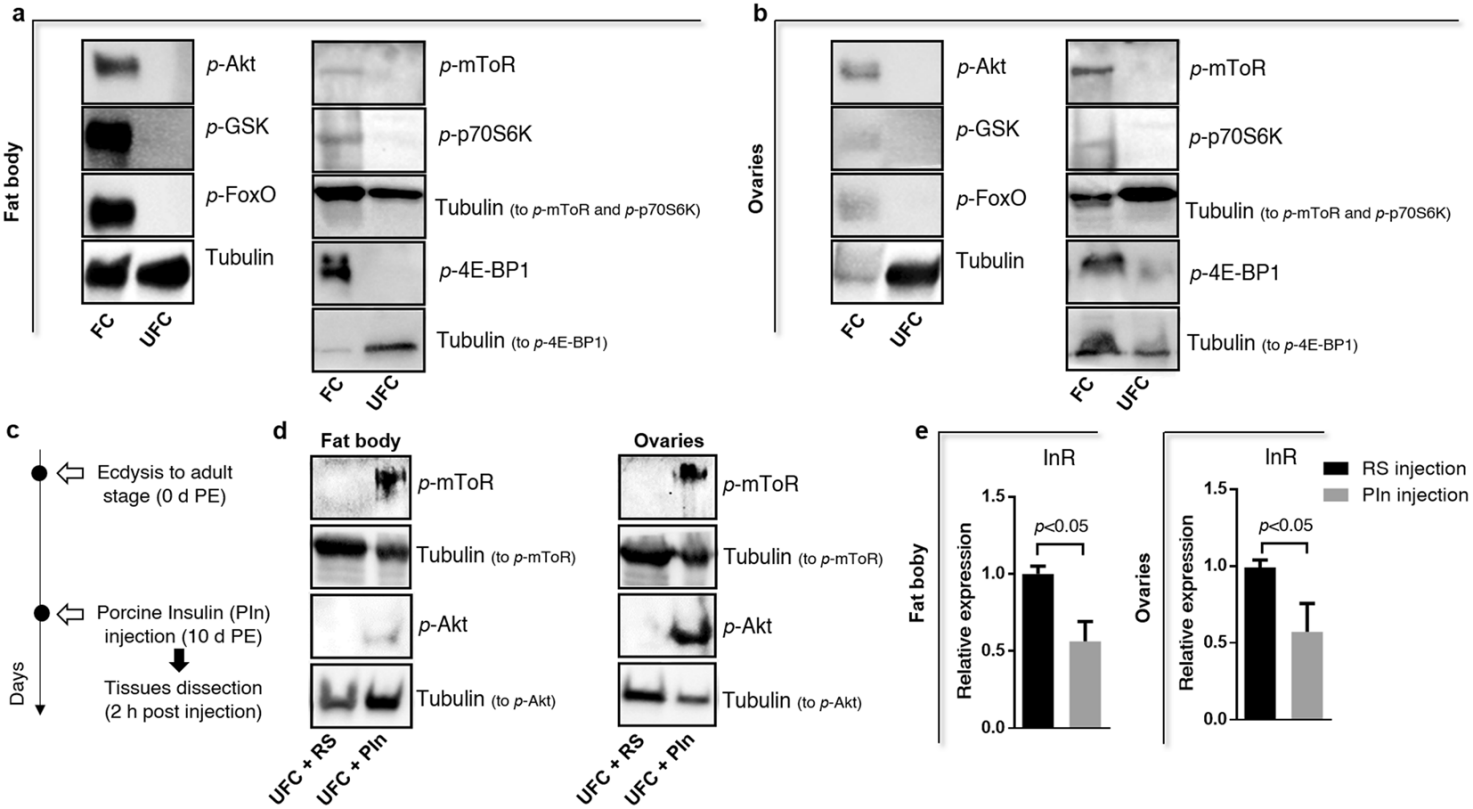
Phosphorylation cascade related with ILP/ToR signaling activation in fat body and ovaries. Western blots were conducted to probe for *p*-Akt, *p*-GSK and *p*-FoxO to test ILP signaling and *p-*mToR, *p*-p70S6K and *p*-4E-BP1 to test mToR signaling (primary antibodies, 1:1,000; visualized using Chemiluminescence). Fat bodies **(a)** and ovaries **(b)** were dissected from adult females 10 days post-ecdysis (UFC) and 3 days post-feeding (FC). The phosphorylation of all 6 proteins are part of the downstream pathway stimulated by ILP/ToR signaling and increased significantly after feeding. **(c–e)** ILP/ToR signaling pathway activation by insulin. (**c**) Experimental scheme; (**d**) After injecting unfed insects with porcine insulin (PIn), an increase in the phosphorylation of Akt (*p*-Akt) and mToR (*p-*mToR) is detected with respect to the *R. prolixus* saline (RS) in both fat bodies and ovaries as observed through western blot analysis. For western blot, images shown are representative of 3 independent experiments. Full-length blots are presented in Supplementary Figs. S6–S8. (**e**) By RT-qPCR, there is a decrease in InR expression when unfed insects are injected with PIn. Results are expressed as mean ± SEM of 3 independent experiments. Graphs and statistical tests were performed using GraphPad Prism 7. The statistical significance of the data was calculated using Student's t-test. A *p* value < 0.05 was considered statistically significant.

In the FB, 28 transcripts involved with ILP/ToR signaling were analyzed, and 9 are up-regulated in the UFC (Table 3), including InR, venus kinase receptor (VKR), insulin receptor substrate 1 (IRS1), phosphatase and tensin homolog (PTEN), ribosomal protein S6 kinase (S6) and IGF. In the OV, and in agreement with the findings from the FB, 13 of 28 transcripts related with ILP/ToR signaling are up-regulated in the UFC, including InR, VKR, IRS1, ToR, ribosomal protein S6 kinase (S6K) and IGF (Table 4). To validate this data, different mRNAs involved with this signaling were chosen and their relative transcripts abundance in FB and OV in both nutritional states monitored by RT-qPCR (Fig. 3a and b, respectively). The results are in agreement with the DEG analysis, suggesting that the transcriptome data is accurate. However, it is important to note that an increase in transcript levels does not necessarily mean that the protein is translated and active^14^. Insulin signaling involves phosphorylation and protein–protein interactions to promote a response^8^. In light of this, we performed western blot studies to understand if the ILP/ToR signaling pathway was activated depending on nutritional condition. The results show that phosphorylated proteins involved with signaling, such as *p*-Akt, *p*-GSK, *p*-FoxO, *p*-p70S6K, *p*-ToR and *p*-4E-BP1, are only expressed in both FB and OV during the FC (Fig. 4). The ribosomal protein S6 was the first identified substrate of S6K, an important regulator of cell growth and cell size, and the translation initiation factor eIF4E was the second well-characterized ToR target^9^. By transcriptome analysis our results show that both mRNA are up-regulated in the OV in fed insects (Table 4), which also suggests that ToR signaling is active. In our experimental conditions, following feeding, the oocytes begin to grow rapidly, and 5–6 days after the blood meal female *R. prolixus* begin egg laying (Supplementary Fig. S4). In some insect species, ILP/ToR signaling plays a key role in nutritional signal transduction activating egg development. With respect to the OV, in *D. melanogaster* the ILP/ToR signaling is involved in germinal stem cell proliferation and maintenance, germline growth and in the control of follicle growth^15^. In the desert locust, *Schistocerca gregaria*, ILP/ToR signaling disruption results in the development of small oocytes^16^ as well as in *Tribolium castaneum* loss-of-function studies of several components of the ILP/ToR signaling results in impairment in the maturation of the primary oocyte and defective oocyte growth^17^. Moreover, in *Aedes aegypti* the ILP/ToR signaling is involved in the regulation of lipid droplet accumulation in oocytes^18^. With respect to the FB, in some species there is a stimulatory ILP/ToR signaling effect on vitellogenin (Vg) synthesis, an essential event for egg growth^6,17,19–20^. IGF is highly expressed in the FB (Table 3) of adult female *R. prolixus* but it remains to be demonstrated whether this peptide could acts on Vg synthesis stimulation. Overall, although we demonstrate an increase in the transcript levels of molecules involved in ILP/ToR signaling in unfed insects (Tables 3 and 4), we did not detect phosphorylated proteins in either FB or OV (Fig. 4). In general, the UFC might favour the up-regulation of hormones involved in catabolism, while those involved in anabolism, such as ILPs, would be down-regulated. In agreement with this hypothesis, our results suggest that circulating ILP and IGF levels might be reduced during the UFC and consequently reduce ILP/ToR signaling. In this sense, Vafopoulou and Steel^21^ reported that the release of ILPs in adult *R. prolixus* is nutrient dependent.

**Table 3:**
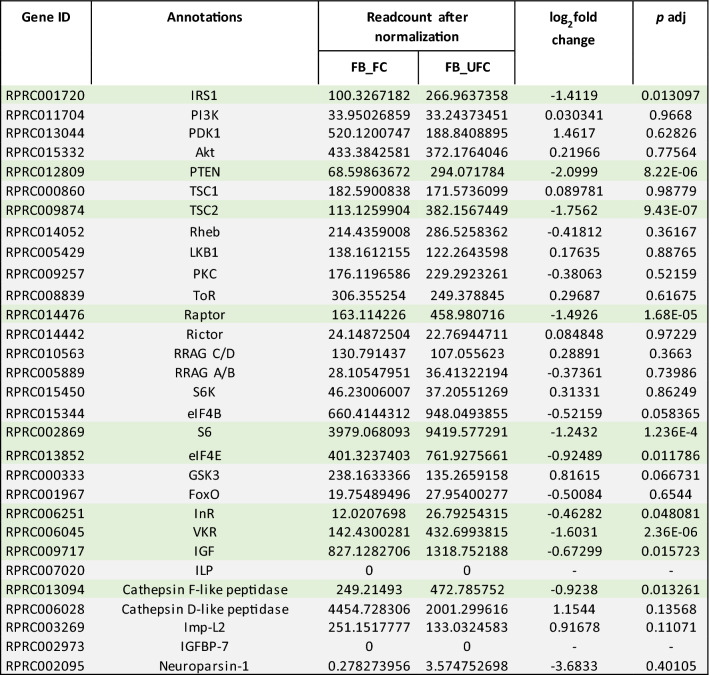
Expression profiles of genes involved in ILP/ToR signaling in fat body under fed and unfed conditions.

*Gray boxes*, genes which are not differentially expressed (non-DEG) between unfed and fed insects; *Green boxes*, DEG up-regulated in unfed insects (UFC). Data shown as log_2_fold change of FC versus UFC. **Gene ID**: VectorBase code (the official gene number in the RproC3 genome assembly); **annotations**: the protein name we are assigning.

**Table 4:**
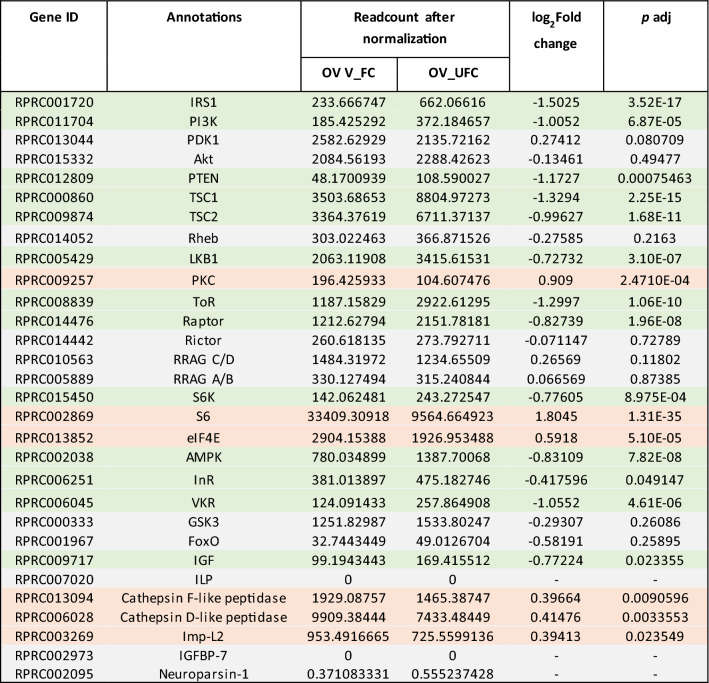
Expression profiles of genes involved in ILP/ToR signaling in ovaries under fed and unfed conditions.

*Orange boxes*, DEG up-regulated in fed insects (FC); *Green boxes*, DEG up-regulated in unfed insects (UFC). *Gray boxes*, genes which are not differentially expressed (non-DEG) between FC and UFC. Data shown as log_2_fold change of FC versus UFC. **Gene ID**: VectorBase code (the official gene number in the RproC3 genome assembly); **annotations**: the protein name we are assigning.

In *A. aegypti* it was demonstrated that starvation significantly increased the mRNA expression of the InR in corpora allata, the gland that synthesizes and releases JH^22^. Also, in *Bombyx mori*, expression of InR, IRS, PI3K, and phosphoinositide dependent protein kinase (PDK) is elevated in the FB when animals cease feeding^23^. Thus, the cells of these tissues could accumulate higher levels of various factors involved in the ILP/ToR signaling, thereby establishing a sensitized state to respond quickly to changes in ILP levels. In this way, when nutrient conditions become favorable, the cells are able to respond rapidly by turning on the mechanisms that stimulate growth. With this in mind, we perform ILP/ToR signaling activation tests by in vivo assays, injecting porcine insulin into unfed insects. By western blot, the results show that phosphorylated Akt and mToR proteins (*p*-Akt and *p*-mToR, respectively) are up-regulated by insulin, demonstrating that unfed insects are able to respond quickly to insulin levels (Fig. 4d). In fifth instar *R. prolixus*, it has been shown that treatment with porcine insulin leads to an increase of *p*-Akt expression in the FB after a half-hour incubation^12^. Also, it is interesting to note that insulin injection into unfed insects decreases the transcriptional expression of InR in both FB and OV (Fig. 4e). Thus, injection of insulin could be simulating a scenario following a blood meal, where the transcripts involved with the ILP/ToR signaling are down-regulated with respect to the UFC.

VKR belongs to the large tyrosine kinase receptor family, as does the InR. This receptor possesses a Venus Fly Trap extracellular module, a bilobate structure that binds small ligands to induce receptor kinase activity^24^. In mosquitoes, ovary ecdysteroidogenic hormone (OEH), a neuropeptide belonging to a family of small cysteine-rich proteins referred to as neuroparsins, triggers phosphorylation of downstream components associated with the insulin signaling pathway via VKR^25^. Here, we show a higher expression in FB and OV of VKR transcripts in unfed female *R. prolixus* (Tables 3 and 4). In addition, the neuroparsins display clear sequence similarities with the N-terminal hormone-binding module of IGF binding proteins (IGFBPs). Based on studies in locusts, it is suggested that neuroparsins may act in vivo by controlling ILP availability^26^. In our experimental conditions, neuroparsin-1 transcript is exclusively present in the CNS of *R. prolixus*, with a comparable level of expression in both nutritional states. Also, for the first time, we report an IGFBP-related protein in *R. prolixus* with high homology with IGFBP-7. It is interesting to note that IGFBP-7 transcript is only found in the CNS, as is the neuroparsin (Table 2). On the other hand, imaginal morphogenesis protein-Late 2 (Imp-L2), the first functionally characterized insulin-binding protein in invertebrates, counteracts insulin signaling in *D. melanogaster* and was reported as an essential factor for starvation resistance^27^_._ It was suggested to be a putative homolog of vertebrate IGFBP-7, but in *R. prolixus* it seems to be present as two different transcripts, maybe with a common precursor, but with different pattern of expression. In *R. prolixus*, we show that Imp-L2-like is expresses in CNS, FB and OV in both UFC and FC but only in the OV V_FC the expression is up-regulated (Tables 2, 3, 4). These results suggests that VKR, neuroparsin, IGFBP-7 and Imp-L2-likes could be working on the regulation of ILP/IGF availability, each one by specific signaling but possible modulating the binding to its receptors. The tumor suppressor PTEN was originally identified as a negative regulator of PI3K signaling, the main sensor of cell growth, metabolism and survival^28^. In *R. prolixus*, *PTEN* mRNA is up-regulated in FB and OV during the UFC, possibly to prevent PI3K activation. GSK3β is described as a key enzyme involved in glycogen metabolism in mammals. GSK3β transcript expression is increased in the FB of *R. prolixus* during vitellogenesis respect to the UFC (Table 3). In *Rhipicephalus (Boophilus) microplus*, it was proposed that the synthesis of GSK3β is up-regulated in the vitellogenic FB to be released and stored by developing oocytes to promote successful oviposition and hatching^29^.

Autophagy, a process which requires the activation of different peptidases, is inherently related to ILP/ToR signaling. For example, in the FB of *D. melanogaster*, the loss of mToR activity induces autophagy which has a protective role as a growth suppressor^30^. Our results show that two transcripts which encode to *serine-type peptidase* (RPRC004789 and RPRC000107) are up-regulated in the FB_UFC (Supplementary Table S1). These type of enzymes are involved in the proteolytic degradation of cellular macromolecules during autophagy^31^. In addition, cathepsin F, a less studied member of the papain-protease family, is ubiquitously expressed in most tissues. Here, we find an up-regulation of transcripts which encodes to a cathepsin F-like peptidase in the FB_UFC (Table 3). The involvement of this peptidase in apoptosis has been reported^32^. In *D. melanogaster* and the silkworm, *Bombyx mori*, two forms of programmed cell death (PCD) in the remodeling of FB are regulated by hormonal and nutritional signals. In both insects, autophagy gradually increases in larval fat body cells during metamorphosis and is followed by apoptotic events^33^. The regulation between PCD and the FB of *R. prolixus* during different nutritional states is unknown, but it is clear that cathepsin F could be working on the development and/or progress of PCD in this tissue. Therefore, the FB could release nutrients during the UFC to help maintain a stable metabolic state when the animal is deprived of nutrient. In addition, *LKB1* mRNA expression is up-regulated in the OV_UFC (Table 4). This transcript encodes a serine/threonine kinase that directly phosphorylates and activates AMPK, a central metabolic sensor^34^. Upon activation, AMPK phosphorylates and activates TSC1/2. In our experiments, the transcripts for *TSC1* and *TSC2* are increased in the OV_UFC (Table 4); the TSC1/2 complex typically results in downstream inhibition of ToR signaling^9^. In *R. prolixus*, mToR signaling inhibition in ovaries could limit pro-growth signals and also induce autophagy, which in turn provides ATP through the recycling of degradation products. Indeed, 4 transcripts which encode to s*erine-type peptidases* are up-regulated in the OV_UFC (RPRC009729, RPRC009383, RPRC004789, RPRC000107; Supplementary Table S1). It is important to highlight that autophagy in OVs of triatomines during starvation has previously been reported^35^. In addition, our results also show an increase of cathepsin D-like peptidase mRNA levels in the FB and an up-regulation in OVs of fed insects (Tables 3 and 4, respectively). In *R. prolixus* it was reported that cathepsin D is stored in the eggs during vitellogenesis^36^ and then takes part in yolk mobilization during embryogenesis^37^. Supporting this finding, in the triatomine *Dipetalogaster maxima*, it was demonstrated that cathepsin D peptidase is synthesized during vitellogenesis by FB and OV as a yolk protein precursor contributing to the total of cathepsin D stored in the oocytes. However, the activity of this peptidase is higher during the unfed condition in both tissues^38^. Overall, these studies indicate the importance of a cathepsin D peptidase in oocytes of triatomines and suggests that this peptidase could be a yolk protein precursor produced by the OV itself in addition to the FB. Also, it has been reported that the specific temporal pattern of cathepsin F expression in fish indicates a specific role for this peptidase in yolk protein processing events occurring during oocyte maturation and/or early embryogenesis^39^. This finding supports our results (cathepsin F-like peptidase expression up-regulated in the OV_FC), thereby identifying a potential new regulator of reproductive processes in *R. prolixus*.

**Figure 5:**
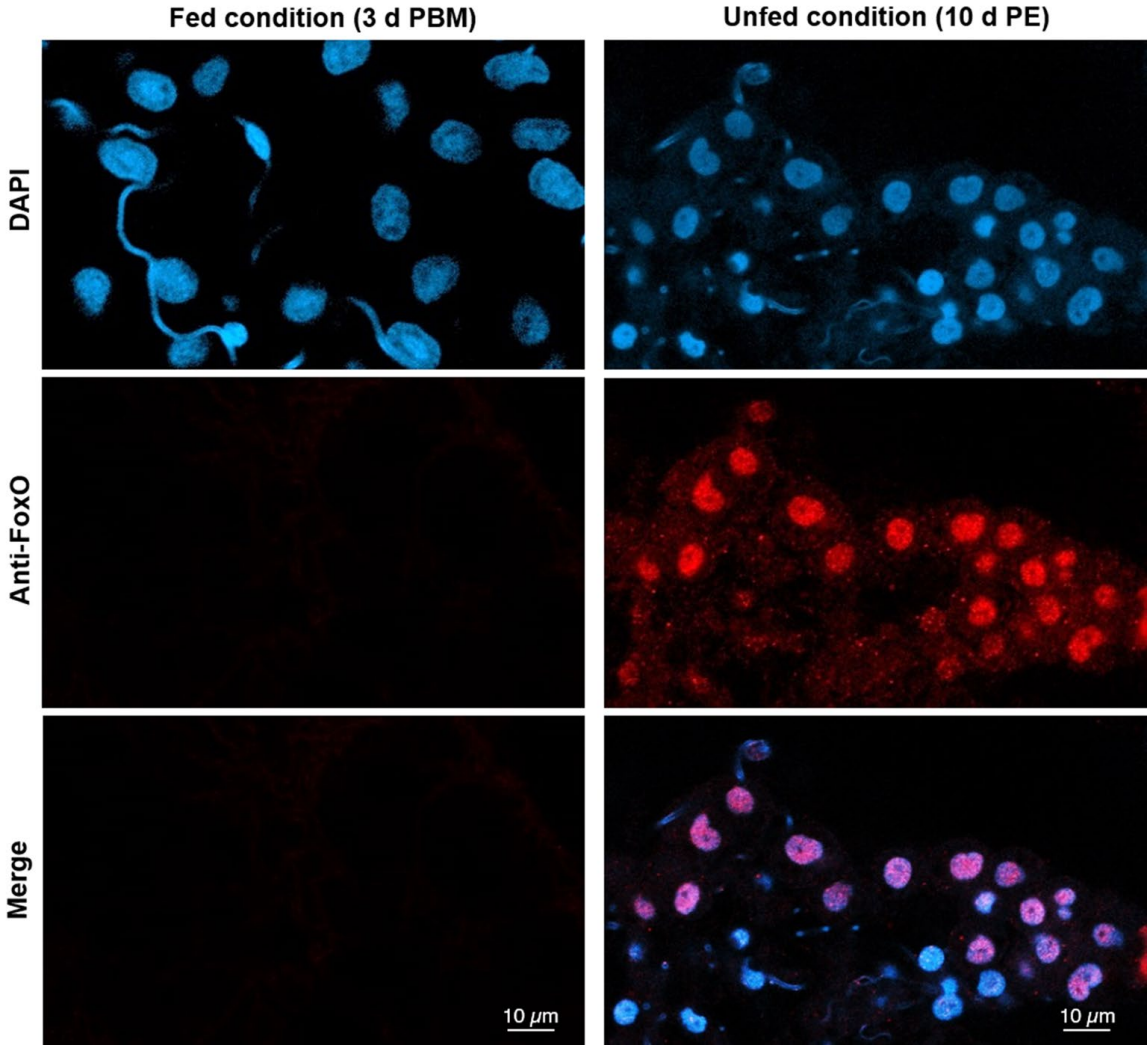
FoxO factor is translocated to nucleus in fat bodies of insects during the unfed condition. Fat bodies from females 10 days after ecdysis (10 days PE) to adult stage and females 3 days post-blood meal (3 days PBM) were incubated with anti-FoxO antibody and processed as stated in “Materials and methods” section. The tissues were then mounted with DAPI and analyzed by scanning laser confocal microscopy. In the merge image, immunofluorescence reveals the co-localization of FoxO (red signal) and DAPI (blue signal) in the nuclei of unfed insects (right panel) but not of fed insects (left panel). Similar results were obtained in 3 independent experiments.

**Figure 6:**
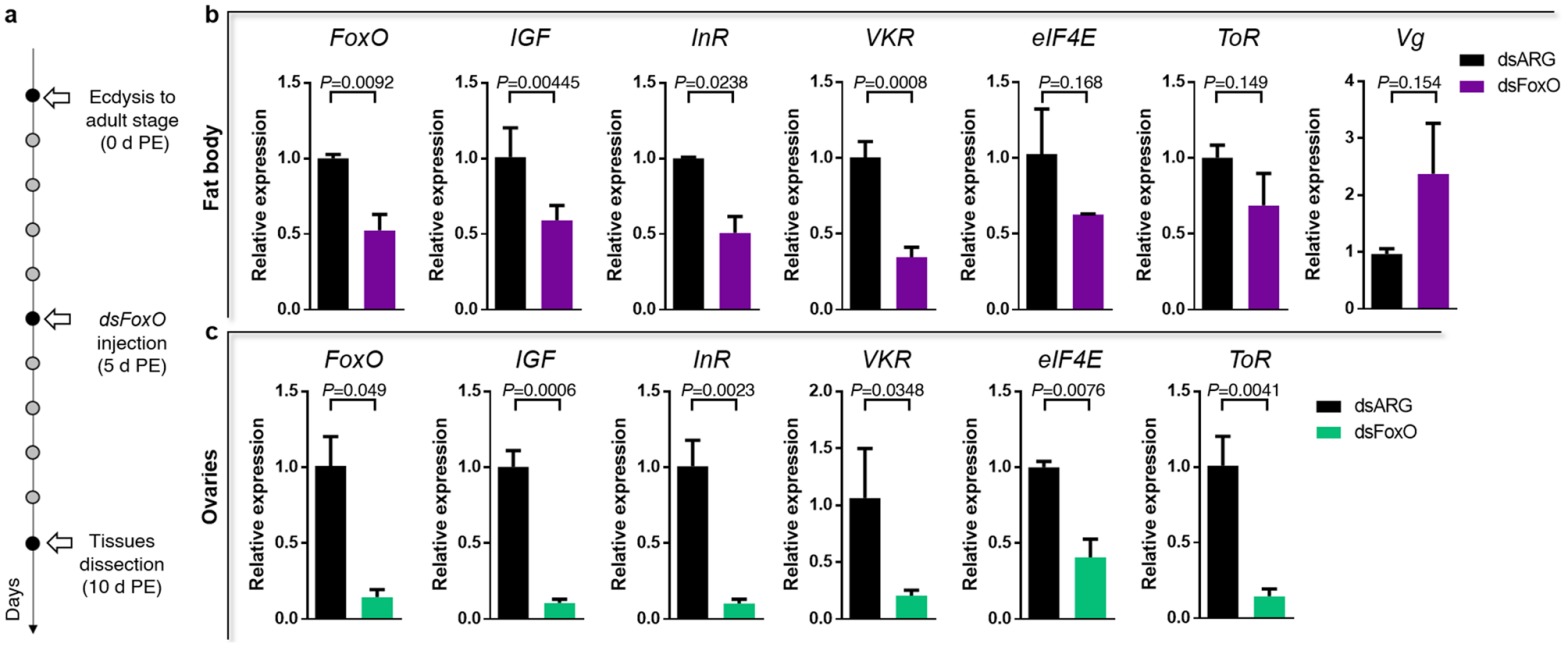
FoxO knockdown in insects during the unfed condition decrease transcript expression of genes associated with ILP/ToR signaling. (**a**) Experimental scheme; (**b, c**) Female in UFC were injected with 5 μL of dsFoxO or dsARG (control). The fat bodies (**b**) and ovaries (**c**) were obtained 5 days post injection and transcript expression of FoxO, IGF, InR, VKR, eIF4E, ToR and Vg measured by RT-qPCR. Values are expressed as mean ± SEM of 3 independent experiments. Graphs and statistical tests were performed using GraphPad Prism 7. The statistical significance of the data was calculated using Student's t-test. A *p* value < 0.05 was considered statistically significant.

ILP/ToR signaling regulates organ size by stimulating cell growth, and thereby increasing cell size but also acts via Akt to inhibit Hippo pathway signaling, which controls organ size by restricting cell number via inhibition of proliferation and induction of apoptosis^40^. KEGG analysis reveals up-regulation of Hippo signaling in both FB and OV during the UFC in *R. prolixus* (Tables 5, 6), reinforcing the hypothesis that ILP/ToR signaling is not active in this state. In *R. prolixus,* this signaling may drive the “organ size checkpoint” controlling the volume of both tissues under low nutritional conditions. Also, by KEGG enrichment, we see an increase in Longevity regulating pathway during the UFC (Tables 5, 6). One target of this pathway is FoxO signaling, which has been described to have pleiotropic effects including those related to stress resistance, metabolism, cellular differentiation and apoptosis^41^. Here, KEGG enrichment show that FoxO signaling is present in FB and OV during the UFC. It was reported in *Blattella germanica* that FoxO transcript expression is not nutritionally regulated^42^ and in accordance, our results indicate that *FoxO* transcript levels remain constant under the different nutritional states in both FB and OV (Tables 3, 4). It is widely known that Akt phosphorylates FoxO (*p*-FoxO) in the cytoplasm, inhibiting its nuclear translocation^41^. For that, FoxO signaling could represent a pathway that indirectly indicates that insulin signaling is absent. By western blot, ours results show that in unfed insects, *p*-FoxO expression is not detected (Fig. 4), promoting the hypothesis that non-phosphorylated FoxO is translocated to the nucleus during the UFC. Using immunofluorescence we found that FoxO staining (red signal) is co-localized with DAPI staining (blue signal) in the nucleus of numerous cells of the FB_UFC but not of the FB_FC (Fig. 5). Control experiments were carried out by omitting one of the antibodies (primary or secondary) and no fluorescence signal was detected (Supplementary Fig. S5). In this sense, FoxO could activate or repress the transcription of a wide array of genes, probably in a tissue-specific manner. In *D. melanogaster*, transcriptional activation of genes by FoxO is a critical step in maintaining survival during amino acid withdrawal^43^. In this scenario, one of the events that depends on FoxO signaling during starvation is the promotion of InR expression^44^. To check if FoxO is responsible for increasing transcript levels involved in ILP/ToR signaling during the UFC in *R. prolixus*, we interfered FoxO signaling in unfed insects by RNAi treatment. The transcripts related to nutritional signaling decrease in unfed *R. prolixus* treated with dsFoxO with respect to the control (dsARG) (Fig. 6). Also, in the same insects, Vg levels in the FB tend to increase with the same treatment. These results suggest that effectively, during the UFC, FoxO is involved in both the insulin sensitivity pathway and the repression of Vg expression. In *B. germanica*, RNAi against FoxO produced a large induction of Vg transcripts expression, which was also reflected in an increase of Vg protein in the haemolymph^42^. In addition, in this cockroach, it has been suggested that the starvation-induced activation of FoxO stimulates the transcription of different genes related to catabolic processes, basically genes involved in lipolysis, glycogenolysis and gluconeogenesis^45^. Furthermore, in the beetle *T. castaneum,* expression of Vg genes is under the control of FoxO regulated by the InR/PI3K pathway^18^. Overall, these results confirm that FoxO signaling is involved in the regulation of different pathways aimed at maintaining the survival of the insect in a specific metabolic state allowing them to respond quickly to the next food supply using ILP/ToR signaling activation.

**Table 5:** The main KEGG pathways in the fat body of *R. prolixus* during different nutritional conditions by transcriptome exploration. The analysis was performed using KEGG database^51^. Statistic method: hypergeometric test.

KEGG pathway up-regulated in fat body of adult fed females	Input number	*p *value
Metabolic pathways	188	2.01E−50
Protein processing in endoplasmic reticulum	42	4.19E−17
Biosynthesis of amino acids	20	6.88E−08
Oxidative phosphorylation	25	7.66E−06
DNA replication	11	9.09E−05
Protein export	9	0.00038982
Endocytosis	18	0.00101893
Spliceosome	16	0.00594405
Insect hormone biosynthesis	4	0.0277457
ABC transporters	3	0.03754539
Terpenoid backbone biosynthesis	4	0.05322105
Carbon metabolism	39	2.20E−16
Citrate cycle (TCA cycle)	19	2.71E−09
Fatty acid metabolism	15	1.12E−06
Pyruvate metabolism	14	8.96E−06
Propanoate metabolism	10	1.44E−05
N-Glycan biosynthesis	12	2.54E−05
Pentose phosphate pathway	9	9.71E−05
Glycolysis/gluconeogenesis	19	1.62E−09
Amino sugar and nucleotide sugar metabolism	12	0.0001191
Fatty acid biosynthesis	6	0.00026484
Glycerophospholipid metabolism	12	0.00083685
Fructose and mannose metabolism	7	0.00419219
Biosynthesis of unsaturated fatty acids	5	0.01264358
Fatty acid elongation	4	0.01989639
Metabolism of xenobiotics by cytochrome P450	8	0.043526173

KEGG pathway up-regulated in fat body of adult unfed females	Input number	*p *value
Ribosome	56	1.07E−15
RNA transport	29	1.71E−07
mRNA surveillance pathway	15	0.00012245
Ribosome biogenesis in eukaryotes	26	0.00028393
mTOR signaling pathway	15	0.00168182
Spliceosome	18	0.001924978
Glycosylphosphatidylinositol(GPI)-anchor biosynthesis	6	0.002805148
Mismatch repair	6	0.003467139
RNA degradation	9	0.0112728
Longevity regulating pathway—multiple species	9	0.01371742
Hippo signaling pathway—fly	7	0.0935007
FoxO signaling pathway	7	0.10548362

**Table 6:** The main KEGG pathways in the ovary of *R. prolixus* during different nutritional conditions by transcriptome exploration. The analysis was performed using KEGG database^51^. Statistic method: hypergeometric test.

KEGG pathway up-regulated in ovaries of adult fed females	Input number	*p* value
Metabolic pathways	357	6.24E−56
Oxidative phosphorylation	102	3.27E−32
Ribosome	105	6.62E−21
Protein processing in endoplasmic reticulum	66	2.74E−16
Spliceosome	56	1.75E−11
RNA transport	55	1.03E−09
Proteasome	31	1.89E−09
Phagosome	30	7.81E−06
Terpenoid backbone biosynthesis	12	0.00022627
Biosynthesis of amino acids	23	0.00022698
Endocytosis	35	0.00030533
Lysosome	27	0.00110357
SNARE interactions in vesicular transport	10	0.00212988
RNA polymerase	12	0.00277896
Notch signaling pathway	9	0.00966772
mToR signaling pathway	22	0.0209
Insect hormone biosynthesis	5	0.087412
N-Glycan biosynthesis	26	3.86E−09
Fatty acid metabolism	21	9.87E−06
Fatty acid elongation	8	0.00226646
Amino sugar and nucleotide sugar metabolism	15	0.0047441
Glycolysis/gluconeogenesis	16	0.00784527
Glycerophospholipid metabolism	15	0.03197737
Other types of O-glycan biosynthesis	5	0.04733204

KEGG pathway up-regulated in ovaries of adult unfed females	Input number	*p* value
AGE-RAGE signaling pathway in diabetic complications	16	0.00055698
ECM-receptor interaction	10	0.00066206
FoxO signaling pathway	22	0.00066206
MAPK signaling pathway—fly	27	0.00107507
Longevity regulating pathway—multiple species	19	0.00240882
Mismatch repair	10	0.00668375
Hippo signaling pathway—fly	18	0.00713181
mTOR signaling pathway	24	0.0071503
Ubiquitin mediated proteolysis	23	0.01368063
RNA transport	30	0.01386011
Fatty acid biosynthesis	6	0.0162938

**Figure 7:**
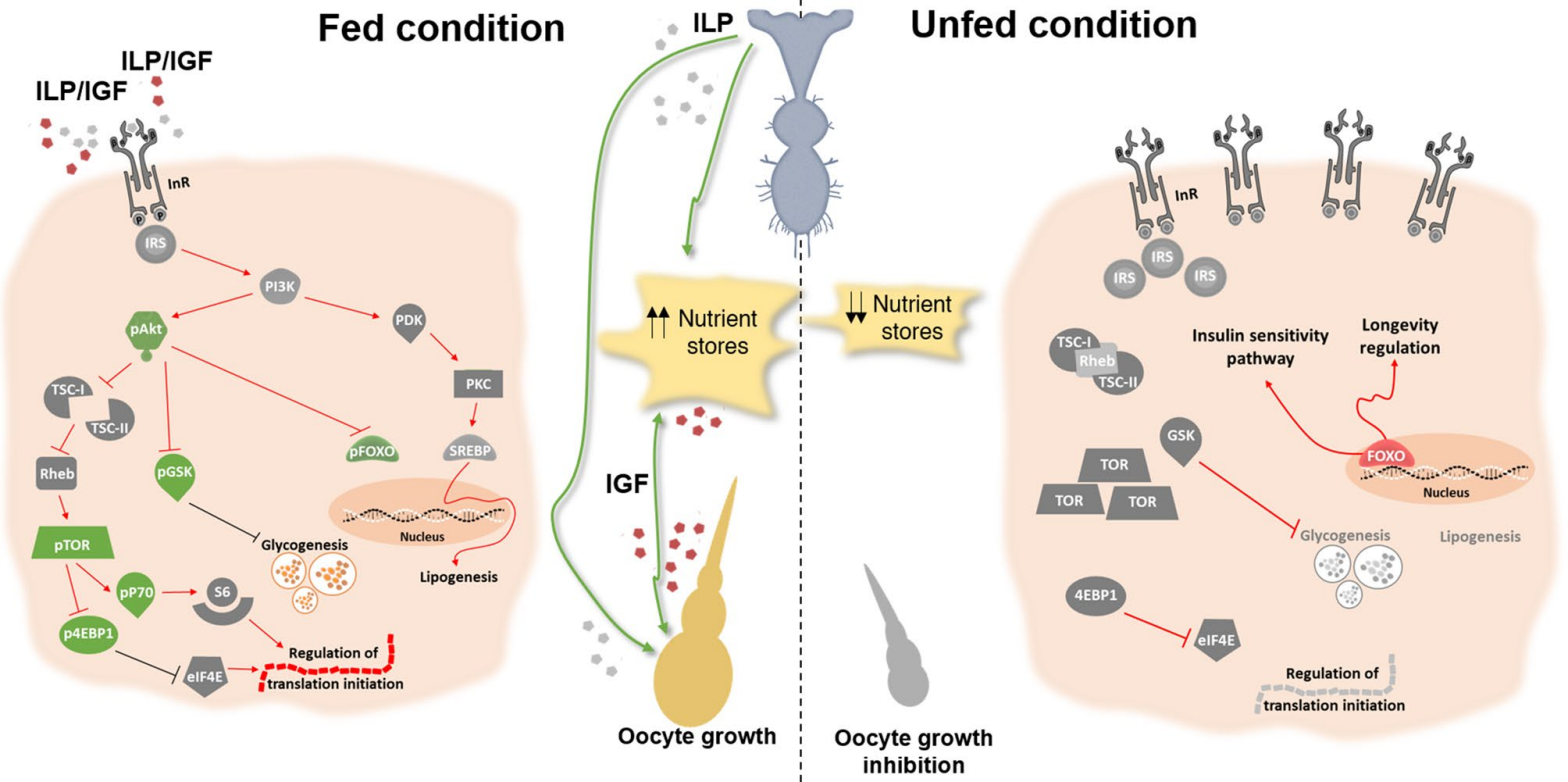
Model of regulatory pathways involved in female reproductive physiology in *Rhodnius prolixus*: impact of the nutritional states. In *R. prolixus*, after a blood meal, ILP/IGF are release into the circulation and promote a phosphorylation cascade mediating the InR signaling activation. By western blot we showed that *p*-Akt, *p*-GSK, *p*-FoxO, *p*-mToR, *p*-p70S6K and *p*-4E-BP1 are up-regulated in both fat body and ovaries (green boxes) of fed females. The presence of other components of the ILP/ToR siganling pathway (gray boxes) were checked by transcriptome analysis or RT-qPCR. In the fed condition, we assume that all of them are actively participating in the signaling cascade, promoting lipogenesis, glycogenesis and translation regulation, along with other events. We suggest that the network of interactions and regulations informed by the nutritional state of the CNS, fat body and ovary, including those related with nutrient biosynthesis and storage, stimulate oocyte growth. In the unfed condition, we demonstrated that in the fat body and the ovaries there is an increase in gene expression of molecules related to the ILP/ToR signaling pathway. However, it is not possible to find these proteins activated (phosphorylated proteins). In unfed females, we suggest that nutrient stores decrease in both tissues and the oocyte growth is inhibited. In addition, by immunofluorescence and gene silencing assays, we show that FoxO factor is translocated to the nucleus in this state and assume that this player a role in increasing the insulin-sensitive pathway and modulating longevity signaling.

Overall, we propose a model for the role of ILP/ToR signaling pathway involved in nutritional states that have effects on reproductive performance (Fig. 7). This research is an important foundation to understanding physiological processes that orchestrate overall reproductive success, as well as the mechanisms involved in maintaining an optimal metabolic state during starvation. It is also important to keep in mind that this work identifies several starting points for new investigations, not only to answer fundamental biological questions but also to advance the development of tools for bio-rational insect control and/or methods for species conservation.

## Materials and methods

### Insects

Adult insects of *R. prolixus* were obtained from an established colony at the University of Toronto Mississauga. Insects were reared in incubators at 25 °C under high humidity (~ 50%). The insects were fed through an artificial feeding membrane as described previously^46^ on defibrinated rabbit blood (Cedarlane Laboratories Inc., Burlington, ON, Canada). For all experiments, males and females, during the last nymphal instar (fifth stage), were separated and fed 30 days post-ecdysis from fourth instars. Insects that gorged at least nine times their own initial body weight (a typical blood meal for fifth instars) were chosen and allowed to molt into the adult stage. Newly-emerged adult females were segregated individually and placed together with a recently fed male. Mating was verified by examining the cubicle for the deposited spermatophore. After copulation, females were fed with a blood meal 10 days post-ecdysis to promote egg growth. Only insects that fed 2.5 to 3 times their initial body weight (a typical blood meal for adults) were used to experiments. CNS, fat body (FB) and ovaries (OV) were sampled from adult females on representative days of the unfed condition (UFC) and fed condition (FC): (a) 10 days post-ecdysis as UFC and (b) 3 days post-feeding as FC (vitellogenesis). In supplementary Fig. S4, the FB and OV morphologies in both nutritional states can be observed. Briefly, triatomine ovarioles are composed of a tropharium containing nurse cells and a vitellarium containing oocytes or follicles in different development stages^4^. The nurse cells transport nutrient to immature oocytes (β and ϒ oocytes or pre-vitellogenic oocytes). After an appropriate nutritional stimulus, the follicular cells surrounding the mature oocytes (α oocytes or vitellogenic oocytes) shrink, a phenomenon called patency, generating an enlargement of interfollicular channels to allow for macromolecules from the hemolymph to be taken up by oocytes^4^. The ovarioles from insects during the FC were further separated according to Brito et al.^47^: (a) pre-vitellogenic ovariole (OV PV_FC), which include the tropharium and immature oocytes, and (b) vitellogenic ovariole (OV V_FC), which are the follicles containing mature oocytes. Ovarioles during UFC were used in their entirety (OV_UFC) (Supplementary Fig. S4).

### Indirect immunofluorescence, DAPI and phalloidin-TRITC staining

The tissues for indirect immunofluorescence, DAPI or phalloidin-TRITC staining were dissected under *R. prolixus* saline (NaCl 150 mM, KCl 8.6 mM, CaCl_2_ 2.0 mM, MgCl_2_ 8.5 mM, NaHCO_3_ 4.0 mM, HEPES 5.0 mM, pH 7.0) and immediately fixed in 4% paraformaldehyde in phosphate buffered saline (PBS, 6.6 mM Na_2_HPO_4_/KH_2_PO_4_, 150 mM NaCl, pH 7.4) at room temperature for 1 h. For immunofluorescence, the tissues were incubated at room temperature for 1 h in 4% Triton X-100/10% NGS (Normal Goat Serum, Sigma-Aldrich, ON, Canada)/PBS. After 3 washes with PBS, tissues were incubated with an anti-FoxO rabbit polyclonal antibody (Cell Signaling Technology, MA, USA), diluted 1:100 in 0.4% Triton X-100/2% NGS/PBS, at 4 °C for 48 h. Then, the tissues were washed and incubated in secondary antibody (Alexa Fluor 488 goat anti-rabbit IgG (H + L), 1:700 in 10% NGS/PBS) at 37 °C for 1 h. Two control experiments were performed by omitting one of the antibodies, primary antibody (anti-FoxO) or secondary antibody. For phalloidin-TRITC staining, after fixing and washing with PBS, tissues were incubated at room temperature for 20 min in 300 ng/ml phalloidin-TRITC (Sigma-Aldrich, ON, Canada). All tissues were mounted in Fluoroshield with DAPI (Sigma-Aldrich, ON, Canada) and observed under a Zeiss laser scanning confocal microscope LSM800, using the LSM image browser software (Carl Zeiss, Jena, Germany). Three independent experiments were performed (n = 3) with each n composed of a pool of 5 tissues.

### RNAseq library preparation

CNS, OV and ventral and dorsal FB of *R. prolixus* females during UFC and FC were dissected in cold autoclave PBS. Three independent experiments were performed (n = 3) with each n composed of a pool of 10 tissues. RNA extraction was performed with Trizol reagent (Invitrogen by Thermo Fisher Scientific, MA, USA) according to manufacturer's instructions. RNA samples were subjected to DNase treatment (Millipore-Sigma, WI, USA) and then repurified with PureLink RNA Mini Kit (Ambion by Thermo Fisher Scientific, MA, USA). RNA integrity and quantification were assessed using the RNA Nano 6000 Assay Kit with an Agilent 2,100 Bioanalyzer system (Agilent Technologies, CA, USA). Libraries for sequencing were generated using NEBNext Ultra RNA Library Prep Kit for Illumina (New England Biolabs, MA, USA) following manufacturer’s recommendations. A total amount of 1 µg RNA per sample was used as input material for the reverse transcription. The libraries were sequenced on Illumina HiSeq platforms (*HiSeq *2500) at the Novogene sequencing facility (California, USA).

### Bioinformatic analyses

The present work was analyzed using gene annotation from the RproC1.3 gene set (ftp://ftp.ensemblgenomes.org/pub/metazoa/release-42/gff3/rhodnius_prolixus/Rhodnius_prolixus.RproC3.42.gff3.gz), and *Rhodnius prolixus* alternative annotation—gene set^48^. FASTX-Toolkit (https://hannonlab.cshl.edu/fastx_toolkit/) was used to filter and trim sequences based on quality. HISAT2 was selected to map the filtered sequenced reads to the reference genome. In order to analyze gene expression levels, Fragments Per Kilobase of transcript sequence per Millions base pairs sequenced (FPKM) was used. HTSeq v0.6.1 software was performed to analyze the gene expression levels. To compare total gene expression levels in CNS, FB and OV during different nutritional conditions, violin plots were used. Venn diagrams were performed to analyse the number of genes that were uniquely expressed within each sample with the number of genes that were expressed in two or more samples. To infer the differentially expressed genes (DEG) with good statistical power, DESeq R package software (1.18.0) was used to normalize. DESeq provides statistical routines for determining differential expression in digital gene expression data using a model based on the negative binomial distribution. The resulting P-values were adjusted using the Benjamini and Hochberg’s approach for controlling the false discovery rate. Genes with *p*-adj (*p*-value after normalization) < 0.05 were assigned as differentially expressed. The results are shown as log_2_fold change: log_2_(FC/UFC). Volcano plots were performed to infer the overall distribution of DEG. We used KOBAS software to test the statistical enrichment of differential expression genes in KEGG (Kyoto Encyclopedia of Genes and Genomes) pathways. KEGG enrichment with corrected *p*-values < 0.05 were significantly enriched in DEGs.

### Quantitative real-time PCR (RT-qPCR)

Total RNA was extracted as described above, followed by cDNA synthesis using the High Capacity cDNA Reverse Transcription Kit (Applied-Biosystems, by Fisher Scientific, ON, Canada). RT-qPCR was performed using an advanced master mix with super green low rox reagent (Wisent Bioproducts Inc, QC, Canada). Three independent experiment were performed (n = 3) with each n composed of a pool of 5 tissues. Each reaction contained 3 technical replicates as well as a no template control and a no reverse transcriptase control. The reactions were performed using a CFX384 Touch Real-Time PCR Detection System (BioRad Laboratories Ltd., Mississauga, ON, Canada). Quantitative validation was analyzed by the 2^−ΔΔCt^ method^49^. The primers used (by Sigma-Aldrich, ON, Canada) for amplification are shown in Supplementary Table S2. β-actin, which was previously validated for transcript expression in FB and OV from *R. prolixus* at different nutritional conditions^11,50^, was used as reference genes. The stability test to confirm the use of actin as reference gene in this work is shown in Supplementary Fig. S9. The amplification efficiency for each pair of primers was calculated using standard curves generated by serial dilutions of cDNA. All amplification efficiencies ranged from 96 to 105% for the different pair of primers tested (Supplementary Table S2). For each pair of primers a dissociation curve with a single peak was seen, indicating that a single cDNA product was amplified. Specific target amplification was confirmed by automatic sequencing (Macrogen, NY, USA).

### Western blot assays

Ovaries and ventral and dorsal FB were dissected from insects during UFC and FC under cold *R. prolixus* saline. Three independent experiments were analyzed (n = 3) with each n composed of a pool of 5 tissues. Tissues were immediately submerged in 200 µl of cold, freshly-made lysis buffer (RIPA buffer [150 mM NaCl, 1% Triton X-100, 0.5% sodium deoxycholate, 0.1% SDS, 50 mM Tris, pH 8.0 in double-distilled or MilliQ water] plus protease and phosphatase inhibitor cocktails (Sigma-Aldrich, ON, Canada)) and homogenized. The homogenates were then centrifuged at 4 °C for 25 min and 17,000× g. The resulting infranatant was collected and used for western blotting. Protein quantification was done using the BCA protein quantification assay (Pierce BCA Protein Assay Kit by Thermo Fischer, ON, Canada). Gel electrophoresis conditions were performed according to Defferrari et al.^12^. Protein bands were separated under reducing conditions on 4–15% pre-made (Mini-Protean TGX Stain-Free Precast Gels, BioRad) 6.5% and 12% Tris–Glycine-SDS gels and loaded in equal amounts across all wells (40 μg each one). After electrophoresis, proteins were transferred to a low-fluorescence PVDF (LF-PVDF) membrane in transfer buffer over 3 min, using a Trans-Blot Turbo Transfer System (all reagents/materials: BioRad Laboratories Ltd., ON, Canada). Membranes were then blocked in PBS-T (PBS containing 0.1% Tween-20) and 5% non-fat milk for 1 h at room temperature. After blocking, the blots were incubated overnight at 4 °C, with primary antibody (1:1,000 dilution in PBS-T with 3% BSA): anti-phospho-Akt (ser473); anti-phospho-GSK3β (ser9); anti-phospho-FoxO (ser256); anti-phospho-ToR (ser2448); anti-phospho-p70S6K (ser434); anti-phospho-4E-BP1 (Thr37/46) (all rabbit antibodies from Cell Signaling Technology, MA, USA) and anti-tubulin (mouse monoclonal antibody from Life Technologies, ON, CA). The specificity of these antibodies has been previously reported^12^. Primary antibodies were washed-off with PBS-T followed by incubation in secondary antibody (1:5,000, horseradish peroxidase (HRP)-conjugated anti-mouse or anti-rabbit antibodies, from Cell Signaling Technology) for 1 h at room temperature with constant agitation. Blots were then washed with PBS-T and visualized using enhanced chemiluminescence (Clarity Western ECL Substrate, BioRad), imaged on a ChemiDoc XRS system and analyzed using Image Lab 5.0 (BioRad software and system).

### Insulin signaling stimulation

Unfed insects were injected with 5 µl of 0.1 µg/µl porcine insulin (Millipore-Sigma, ON, Canada) or 5 µl of *R. prolixus* saline (control). Two hours post injection, FB and OV of the insects were removed and subjected to western blot as previously described.

### Double-stranded RNA design and synthesis

A 161-base pair template was used to synthesize a double stranded RNA molecule (dsFoxO) using the T7 Ribomax Express RNAi System (Promega, WI, USA), according to the manufacturer protocol. Gene specific primers (GSP) were combined with GSPs containing the T7 RNA polymerase promoter sequence (Supplementary Table S2). As an experimental control, a dsRNA molecule based on the Ampicillin Resistance Gene (dsARG) from the pGEM-T Easy Vector system (Promega, WI, USA) was used throughout the study^12^.

### Knockdown of FoxO transcript expression using double stranded RNA

To knockdown the expression of *FoxO*, females 5 days post-ecdysis to adult stage, were injected into the hemocoel with 2 μg of dsARG or dsFoxO in 5 μL of ultrapure water using a Hamilton micro syringe (Hamilton Company, NV, USA). Insects were dissected 5 days post-injection and *FoxO*, *IGF*, *InR*, *VkR*, *elF4E*, *ToR* and *Vg* transcript expressions were measured in FB and OV by RT-qPCR assays, as described above.

## Supplementary information

Supplementary file1

## Data Availability

The raw sequence dataset of this project is registered with the National Center for Biotechnology Information (NCBI) under PRJNA624187 and PRJNA624904 bioprojects.
