# Supplementary material for: Transcriptomic analysis of regulatory pathways involved in female reproductive physiology of Rhodnius prolixus under different nutritional states
Source: Sci Rep. 2020 Jul 10;10:11431. doi: 10.1038/s41598-020-67932-4 (PMC7351778; doi:10.1038/s41598-020-67932-4)
Supplement: Supplementary file 1 — Supplementary file1 [file 41598_2020_67932_MOESM1_ESM.docx]

Transcriptomic analysis of regulatory pathways involved in female reproductive physiology of *Rhodnius prolixus* under different nutritional states

Jimena Leyria^1,^*, Ian Orchard^1^ and Angela B. Lange^1^

^1^Department of Biology, University of Toronto Mississauga, Mississauga, ON, Canada

* Corresponding author: jimenal.leyria@utoronto.ca

**Supplementary Figures S1 to S10 and Supplementary Tables S1 to S2**

**
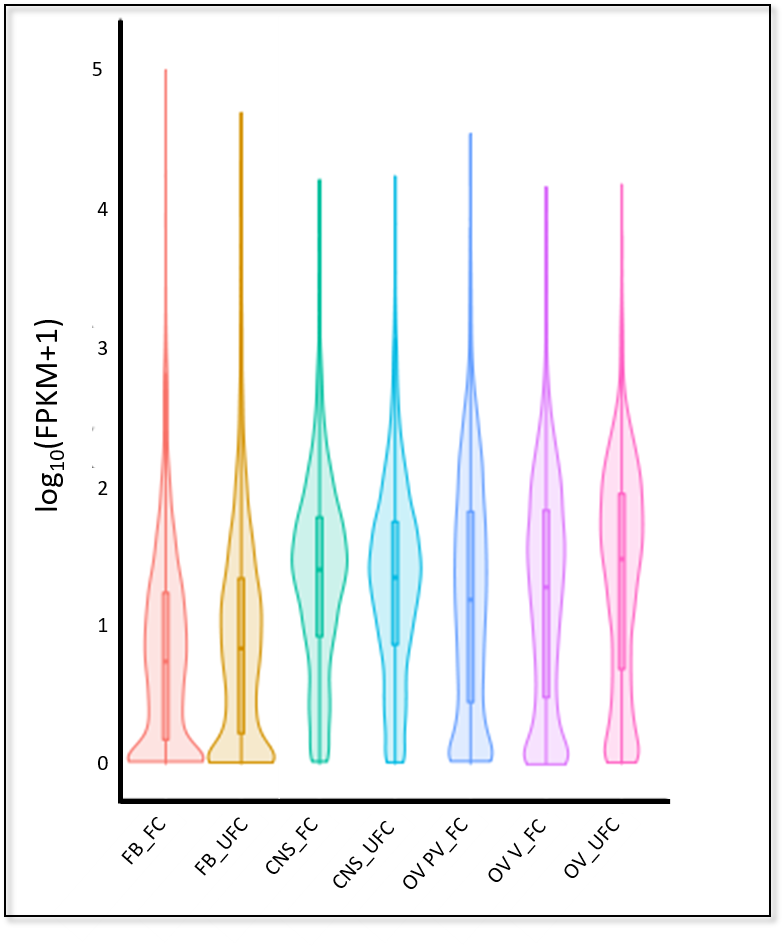
**

**Supplementary Fig. S1. Total gene expression level comparison by violin plot**. The final Fragments Per Kilobase of transcript sequence per Millions base pairs sequenced (FPKM) is the mean value of the biological replicates (n = 3). The x-axis shows the sample names and the y-axis shows the log_10_(FPKM+1). The violin width indicates the gene density.


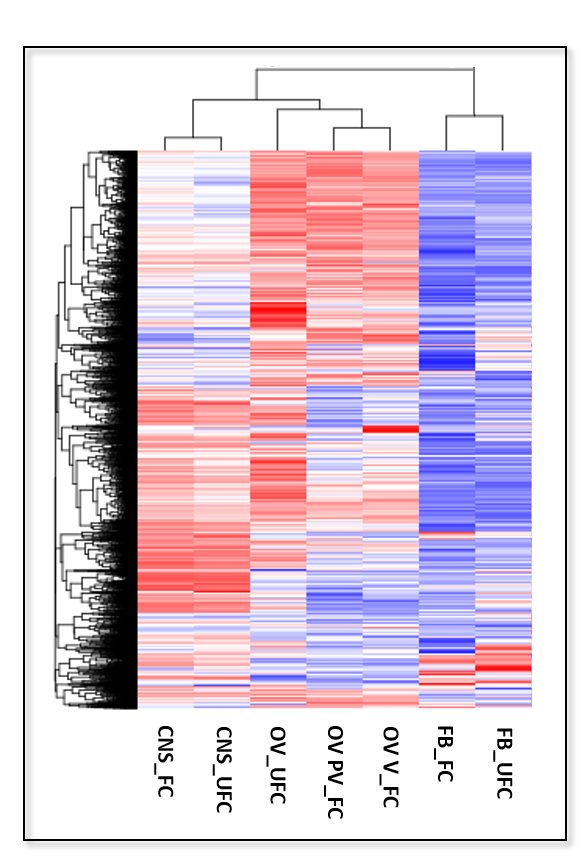


**Supplementary Fig. S2.** **Cluster analysis of gene expression.** The overall results of FPKM cluster analysis, clustered using the log10(FPKM+1) value. Red denotes genes with high expression levels and blue denotes genes with low expression levels. The color range from red to blue represents the the log-10(FPKM+1) value from large to small. The vertical distances on each branch of the dendrogram represent the degree of similarity between gene expression profiles of the different samples


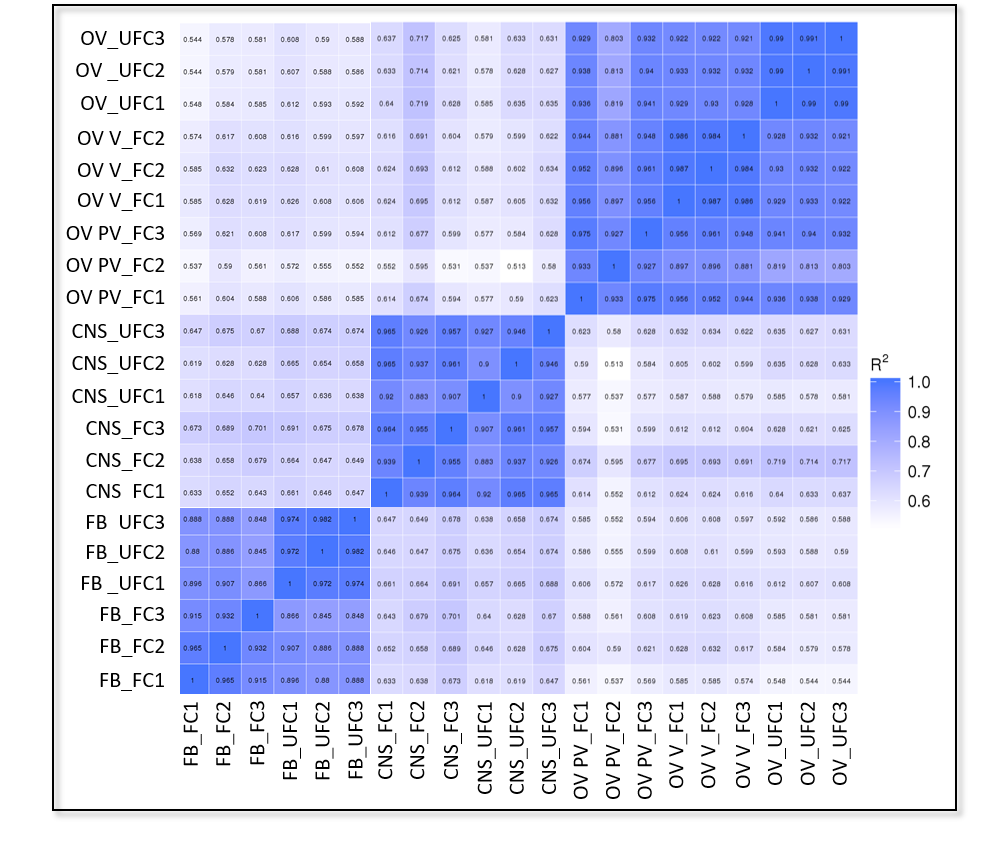


**Supplementary Fig. S3. Heat map of correlation coefficient between biological replicates.** It can be observed in each box, the Pearson correlation coefficient (R^2^) close to 1.


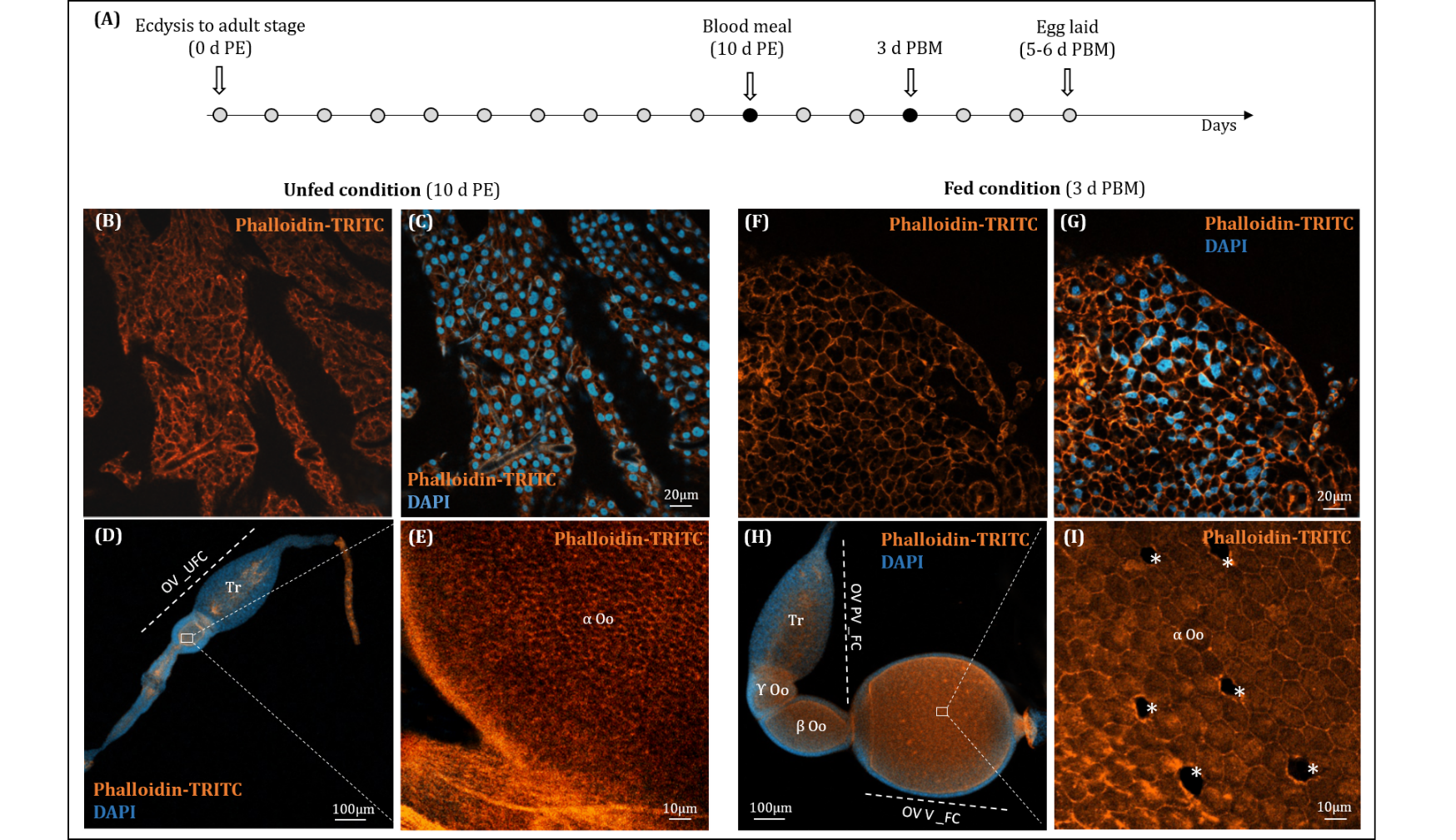


**Supplementary Fig. S4. Morphologies of fat bodies and ovaries of unfed and fed insects.** CNS, fat body and ovaries were sampled from insects at representative days of the unfed condition (10 days post ecdysis (10 d PE)) and fed condition (3 days post-blood meal (3 d PBM)). **(A)** Timeline showing the representative days used in this work; **(B-E)** DAPI and Phalloidin-TRITC staining of a fat body (B-C) and an ovariole (D-E) of an unfed insect. **(F-I)** DAPI and Phalloidin-TRITC staining of a fat body (F-G) and an ovariole (H-I) of a fed insect. Tr, tropharium; ϒ Oo, gamma oocyte; β Oo, beta oocyte; α Oo, alpha oocyte; *, patency; OV PV_FC, pre-vitellogenic ovariole during the fed condition (tropharium and ϒ and β oocytes); OV V_FC, vitellogenic ovariole during the fed condition (follicles containing α oocyte); OV_UFC, ovariole during unfed condition (tropharium and oocytes).


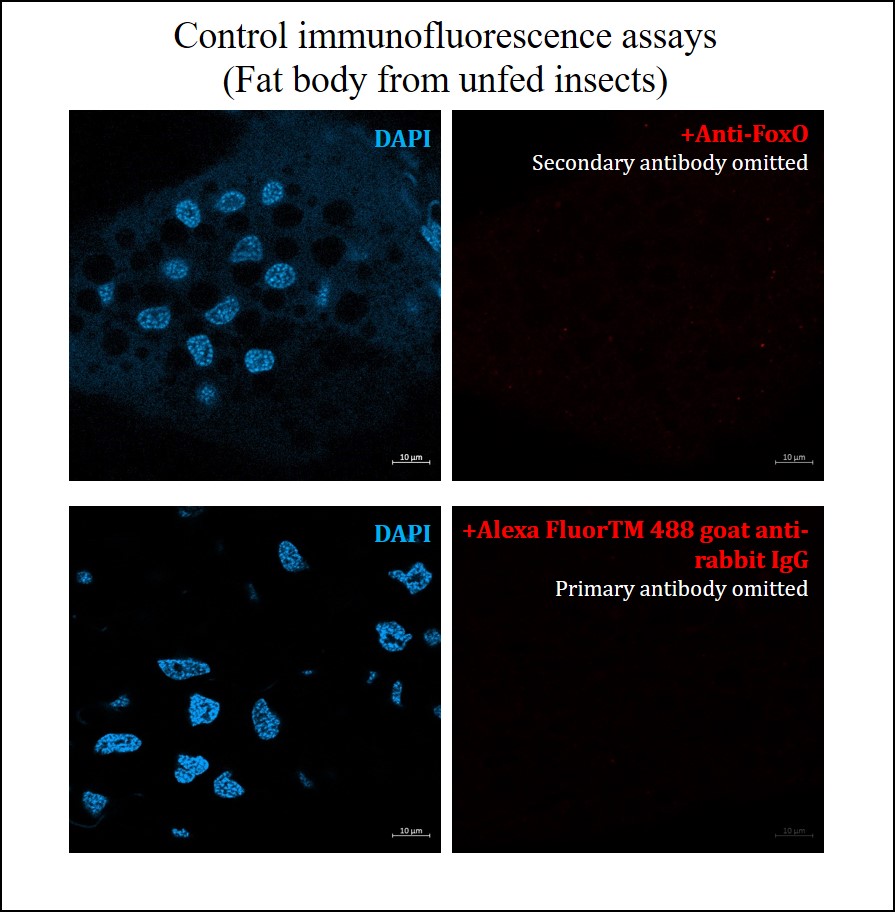


**Supplementary Fig. S5.** Control experiments to immunofluorescence assays (shown in Fig. 5) were carried out by omitting one of the antibodies (primary or secondary). No fluorescence signal was detected.

**
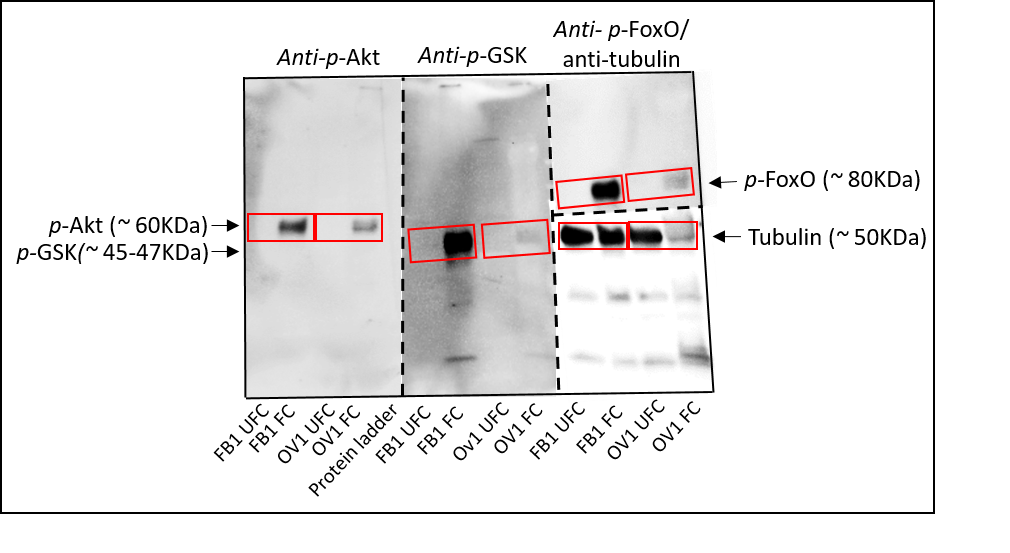
**

**Supplementary Fig. S6. The uncropped images for western blots shown in Figures 4A and B.** Protein extracts from fat body (FB) and ovaries (OV) homogenates (40 μg each one) during the unfed condition (UFC) and fed condition (FC) were subjected to pre-made (4-15 % Mini-PROTEAN™ TGX Stain-Free™ Protein Gels, 15 well, 15 µl) to detect *p*-Akt, *p*-GSK and *p*-FoxO. Images displays chemiluminescent detection to anti-*p*-Akt, anti-*p*-GSK and anti-*p*-FoxO for blots shown in Fig. 4A-B (with its corresponding anti-tubulin blot). Note that the blots were cropped to incubate with the different antibodies (dashed lines). Red boxes display the cropped area used in the main figures; solid lines indicate the borders of the blots. To detect *p*-Akt, *p*-GSK and tubulin, different blots with the samples were used due to the proximity of their molecular weight. In Figure 4A-B these images have been flipped for presentations reasons. The specificity of these antibodies has been previously reported [Defferrari, M. S., Da Silva, S. R., Orchard, I. & Lange, A. B. A *Rhodnius prolixus* insulin receptor and its conserved intracellular signaling pathway and regulation of metabolism. *Front Endocrinol (Lausanne).* **9**, 745 (2018)].


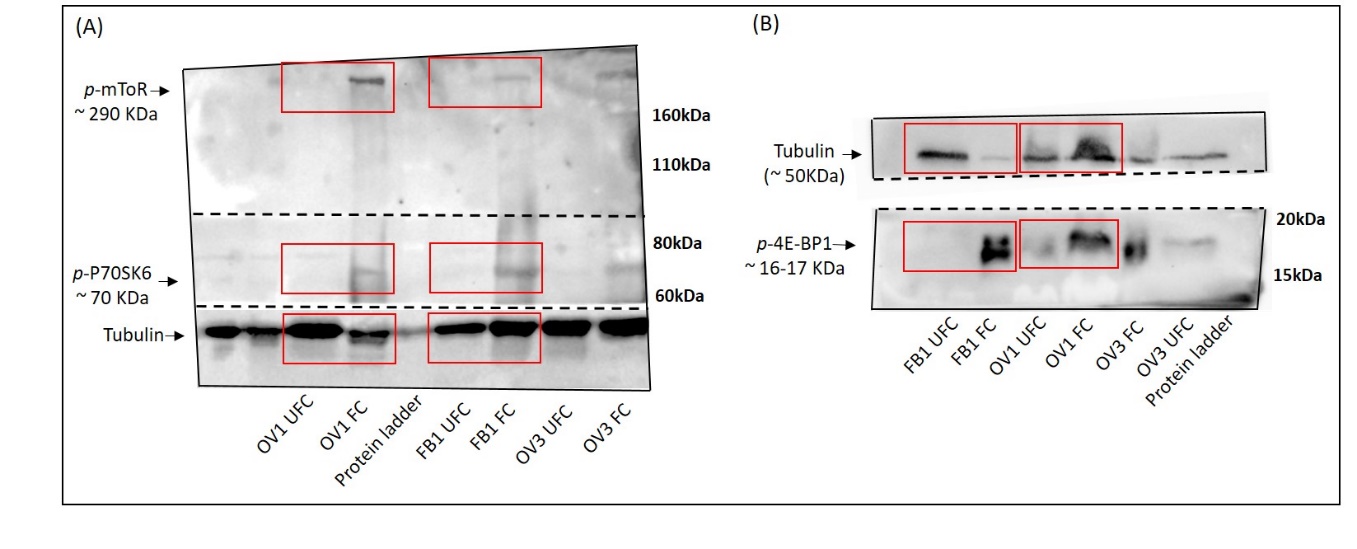


**Supplementary Fig. S7. The uncropped images for western blots shown in Figures 4A and B.** Protein extracts from fat body (FB) and ovaries (OV) homogenates (40 μg each one) during the unfed condition (UFC) and fed condition (FC) were subjected to 6.5 % Tris-glycine-SDS gel to detect *p*-mToR and *p*-p70S6K and to 12 % SDS-Tris-glycine gel to detect *p*-4E-BP1. Images displays chemiluminescent detection to anti-*p*-mToR, anti-*p*-p70S6K **(A)** and anti-*p*-4EBP1 **(B)** for blots shown in Fig. 4A-B (with their corresponding anti-tubulin blots). Note that the blots were cropped to incubate with the different antibodies (dashed lines). Red boxes display the cropped area used in the main figures; solid lines indicate the borders of the blots. In Figure 4A-B these images have been flipped for presentations reasons.


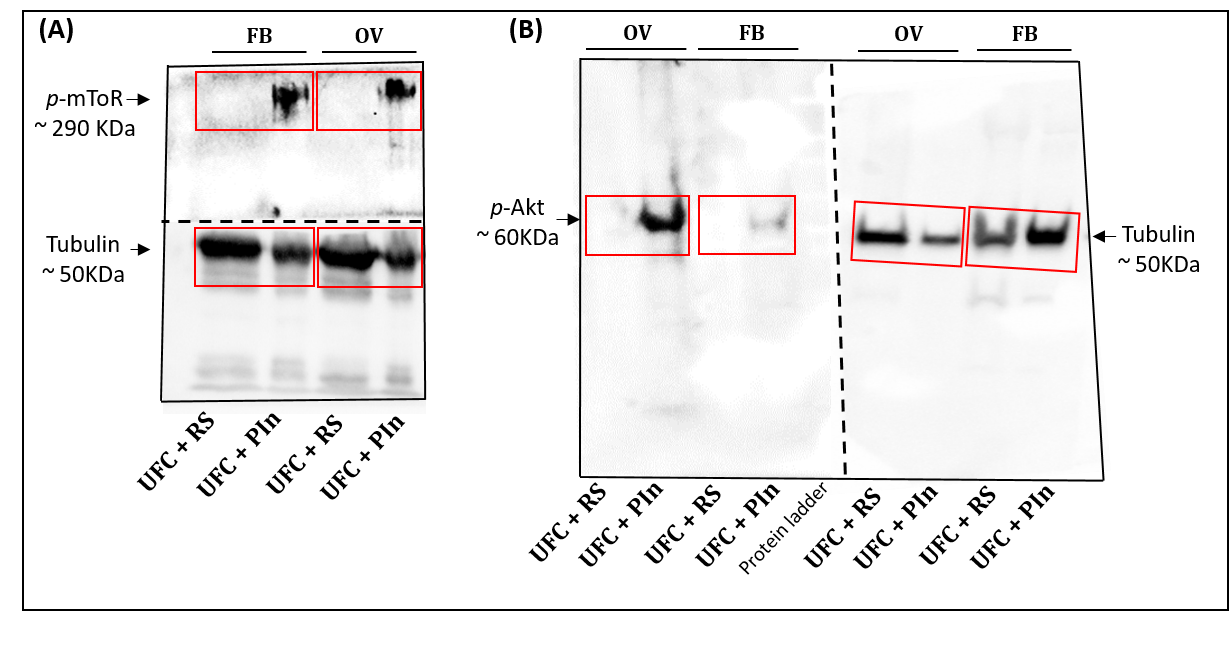


**Supplementary Fig. S8. The uncropped images for western blots shown in Figures 4D.** Protein extracts from fat body (FB) and ovaries (OV) homogenates (40 μg each one) during the unfed condition (UFC) of females post porcine insulin (PIn) or *Rhodnius* saline (RS) injections were subjected to pre-made (4-15 % Mini-PROTEAN™ TGX Stain-Free™ Protein Gels, 15 well, 15 µl) to detect *p*-Akt and 6.5 % Tris-glycine-SDS gel to detect *p*-mToR. Note that the blots were cropped to incubate with the different antibodies (dashed lines). Images displays chemiluminescent detection to anti-*p*-Akt and *p*-mToR for blots shown in Fig. 4D (with its corresponding anti-tubulin blot). Red boxes display the cropped area used in the main figures. Solid lines indicate the borders of the blots. To detect *p*-Akt and tubulin, different blots with the samples were used due to the proximity of their molecular weight.

***
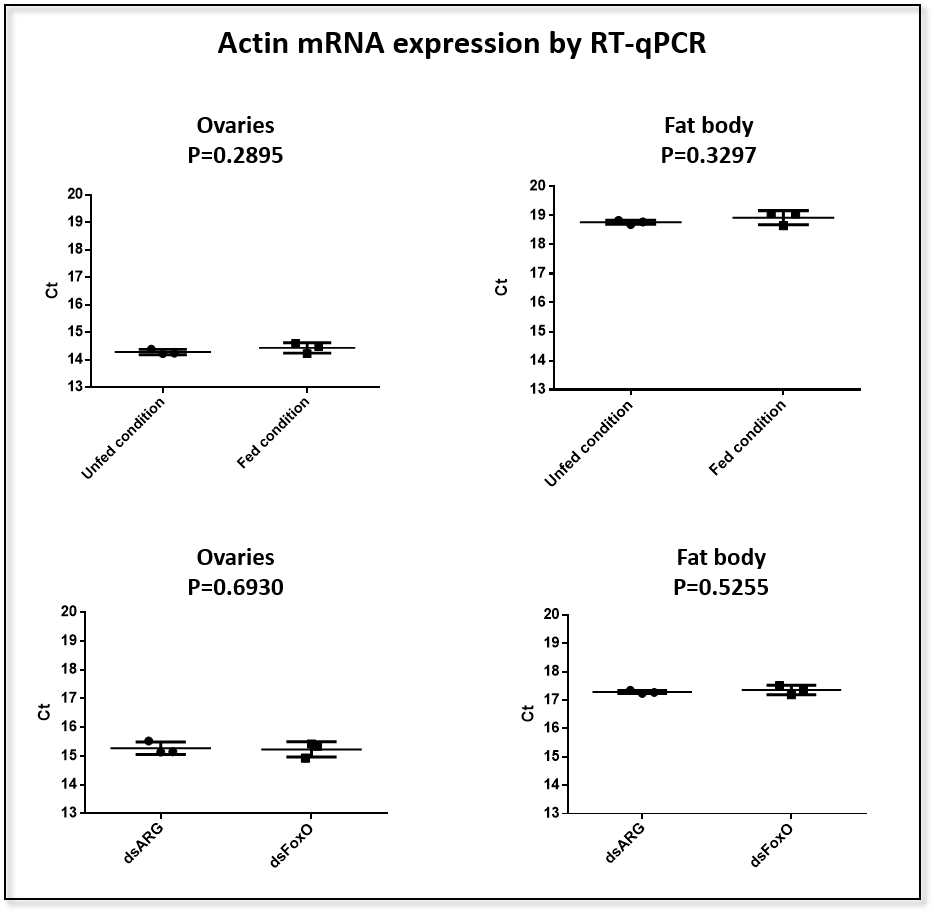
***

**Supplementary Fig S9**. Expression of actin as reference gene in ovaries and fat body of adult female *Rhodnius prolixus*. Quantitative real‐time PCR (RT-qPCR) was carried out using specific primers designed for actin to evaluate the gene stability on tissues under different conditions. Results were plotted as scatter plot of quantification cycle (Ct) values obtained from 3 independent experiments. Statistical analyses were carried out by T-Test.


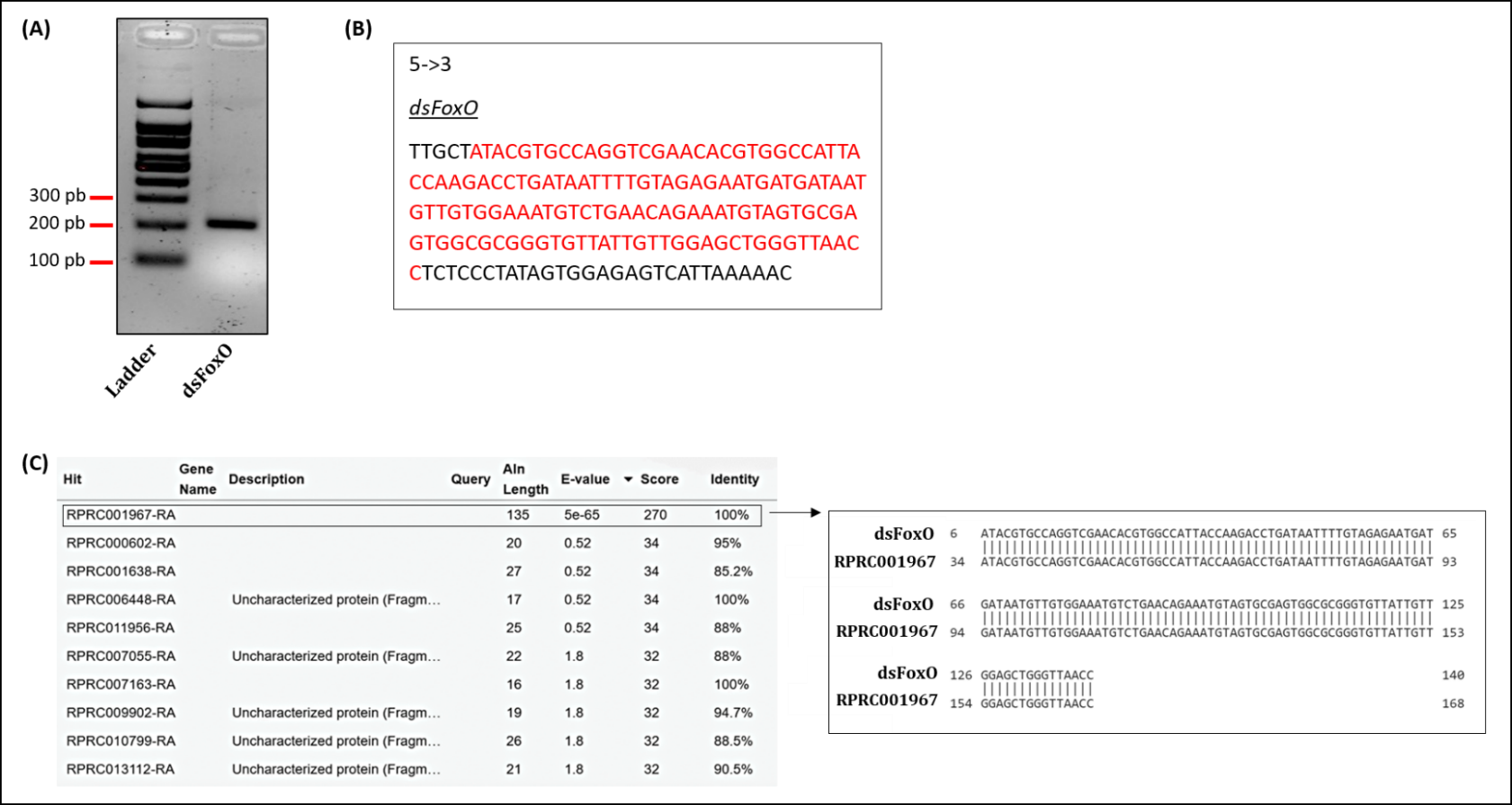
**Supplementary Fig S10**. *DsFoxO* sequencing and specificity. The double-stranded obtained by T7 Ribomax Express RNAi System (Promega, WI, USA) using specific primers (Supplementary Table S2) to dsFoxO, was running in agarose gel (2%). (A) The only band obtained was extracted from gel and the specific target amplification was confirmed by automatic sequencing (Macrogen, NY, USA). (B) Sequence obtained with high quality from Macrogen. (C) The Basic Local Alignment Search Tool (BLAST) was used to compare the sequences. It was performed a pairwise alignment between the sequence obtained from Macrogen and the targets in the database of VectorBase, where *R. prolixus* genome was deposited. Alignments with the best-matching sequences are shown and scored. Only 1 high confidence hits, i.e., genomic regions identified with high similarity with our sequence, was showed. RPRC001967 is the VectorBase code (the official gene number in the RproC3 genome assembly) which encode to FoxO transcription factor. It result confirms the specificity of dsFoxO and validates the experiments using FoxO knockdown in insects.

**Supplementary Table S1. Expression profiles of mRNA which encode to serine peptidases in ovaries and fat body under fed and unfed conditions.**

| **Serine-peptidases** | **VectorBase code** | **OV V_FC** | **OV_UFC** | **log2FoldChange** | ***P*adj** | **FB_FC** | **FB_UFC** | **log2FoldChange** | ***p*adj** |
| --- | --- | --- | --- | --- | --- | --- | --- | --- | --- |
|  | **RPRC010197** | 1215.31708 | 177.946922 | 2.7718 | 2.33E-61 | 2483.466 | 1867.383 | 0.41134 | 0.44371 |
|  | **RPRC014770** | 702.5945173 | 450.516898 | 0.64111 | 0.0002656 | 2578.694 | 513.23 | 2.329 | 6.42E-14 |
|  | **RPRC003090** | 977.3086236 | 490.786879 | 0.99372 | 9.05E-05 | 7473.416 | 2502.589 | 1.5783 | 0.75165 |
|  | **RPRC009729** | 11.17586722 | 29.6644615 | -1.4083 | 0.030312 | 5024.942 | 8597.662 | -0.77484 | 0.3092 |
|  | **RPRC009383** | 127.9790932 | 207.064414 | -0.69417 | 0.0047039 | 7.834 | 5.723 | 0.45299 | 0.9933 |
|  | **RPRC002919** | 146.1151493 | 76.6943964 | 0.92991 | 0.0008258 | 18.15 | 12.592 | 0.52746 | 0.78252 |
|  | **RPRC004789** | 39.462 | 303.46 | -2.943 | 1.54E-29 | 303.055 | 559.022 | -0.88332 | 0.010993 |
|  | **RPRC000033** | 93.272 | 87.2 | 0.097129 | 0.86967 | 816.83 | 1048.282 | -0.35992 | 0.43138 |
|  | **RPRC005405** | 1728.672 | 1701.588 | 0.022783 | 0.97958 | 6051.004 | 8628.186 | -0.51188 | 0.18637 |
|  | **RPRC000107** | 85.8179955 | 203.759506 | -1.2475 | 0.0048602 | 61.373203 | 220.64416 | -1.846 | 0.0000010 |

*Orange boxes*, DEG up-regulated in fed insects (FC); *Green boxes*, DEG up-regulated in unfed insects (UFC). *Gray boxes*, genes which are not differentially expressed (non-DEG) between FC and UFC. Data shown as log_2_fold change of FC vs UFC. VectorBase code: the official gene number in the RproC3 genome assembly)

| Gen | Primer | Sequence (5´-3´) | R^2^ | Efficiency |
| --- | --- | --- | --- | --- |
| InR | InR forward | AGCTCCCAGATTGTCTACGG | 0.9872 | 98.79% |
|  | InR reverse | CCGGGTCGAATCAACTAGG |  |  |
| VKR | VKR forward | CTTCGGATGGTGGGTCTAAA | 0.9992 | 103.94% |
|  | VKR reverse | ACAGCAAATCGATTCCAAGG |  |  |
| Vg | Vg forward | TTGCTAGTCGCATGAACCTG | 0.9928 | 96.1% |
|  | Vg reverse | TTTAGTGGTGCATCGCTCTG |  |  |
| IGF | IGF forward | TGTCATCTCTGCTCCTTTGG | 0.9913 | 96.35% |
|  | IGF reverse | TTGTTATGGCTACCTTTGTCG |  |  |
| Actin | β-actin forward | AGAGAAAAGATGACGCAGATAATGT | 0.9957 | 98.53% |
|  | β-actin reverse | ATATCCCTAACAATTTCACGTTCG |  |  |
| ToR | ToR forward | GGTGCTGGCTCTTCGTACTT | 0.9985 | 104.73% |
|  | ToR reverse | ACAATGTGGAAGGGTCGCAT |  |  |
| Akt | Akt forward | CGAGAAAAAGCCACTGGAAG | 0.9928 | 104.93% |
|  | Akt reverse | CCATAACGAAGCACAGACGA |  |  |
| FoxO | FoxO forward | GCTCTGAGATGGGCCTAGAA | 0.9937 | 105% |
|  | FoxO reverse | GGTTAACCCAGCTCCAACAA |  |  |
| S6K | S6K forward | GGACCCACCCTTTAAACCAT | 0.999 | 103.92% |
|  | S6K reverse | TTTCTTGGCGATCTTGCTTT |  |  |
| S6 | S6 forward | TGAGTGAGGTCTCGTGTTCG | 0.999 | 101.73% |
|  | S6 reverse | CACCGAGTTGATCTGCTTCA |  |  |
| elF4E | eIF4E forward | GGGAAGACGCAGCAAATAAA | 0.9964 | 96.48% |
|  | eIF4E reverse | TTCCTCCCAATTGCTACCAC |  |  |
| GSK | GSK forward | TGCCCCAAAAATAGTTGCTC | 0.9992 | 104.89% |
|  | GSK reverse | TGTGAAGCAGCTCTCTCCAA |  |  |
| dsFoxO | dsFoxO forward | **TAATACGACTCACTATAGGGAGA**GCTCTGAGATGGGCCTAGAA |  |  |
|  | dsFoxO reverse | **TAATACGACTCACTATAGGGAGA**GGTTAACCCAGCTCCAACAA |  |  |

**Supplementary Table S2.** Primers list.

***TAATACGACTCACTATAGGGAGA =** T7 RNA polymerase promotor
